# Supplementary material for: Effect of rising fuel prices on small-scale fisheries livelihoods and marine sustainability in Ghana
Source: PLoS One. 2025 Jan 13;20(1):e0317260. doi: 10.1371/journal.pone.0317260 (PMC11729924; doi:10.1371/journal.pone.0317260)
Supplement: S1 Table — (DOCX) [file pone.0317260.s001.docx]

**S1_Table****.docx**

| Variable | Value | APAM(N=150) | WINNEBA(N=170) |
| --- | --- | --- | --- |
| AGE | BELOW 20 | 6(3.8) | 10(5.9) |
|  | 20-39 YEARS | 66(44.2) | 73(43.1) |
|  | 40-59 YEARS | 58(38.5) | 63(37.3) |
|  | ABOVE 60 | 20(13.5) | 24(13.7) |
| MARITAL STATUS |  |  |  |
|  | SINGLE | 26(17.3) | 50(29.4) |
|  | MARRIED | 89(59.6) | 77(45.1) |
|  | DIVORCED | 21(13.5) | 23(13.7) |
|  | SEPARATED | 14(9.6) | 20(11.8) |
| EDUCATIONAL LEVEL |  |  |  |
|  | NO FORMAL EDUCATION | 65(43.2) | 49(28.8) |
|  | ELEMENTARY | 56(37.2) | 85(50.0) |
|  | SECONDARY | 26(17.6) | 36(21.2) |
|  | TERTIARY | 0(0.0) | 0(0.0) |
|  | TECHNICAL TRAINING | 3(2.0) | 0(0.0) |
| TYPE OF FISHER |  |  |  |
|  | CAPTAIN | 38(25.0) | 37(21.6) |
|  | CREW MEMBER | 61(40.4) | 70(41.1) |
|  | BOAT/CANOE OWNER | 51(34.6) | 63(37.3) |
| NUMBER OF PEOPLE IN YOUR HOUSEHOLD | LESS THAN 5 | 20(13.4) | 27(15.7) |
|  | 6 - 10 | 92(61.5) | 87(51.0) |
|  | MORE THAN 10 | 38(25.1) | 36(25.1) |
| WHAT IS YOUR AVERAGE MONTHLY INCOME FROM FISHING? | 0 - 199 | 0(0.0) | 0(0.0) |
|  | 200 - 399 | 9(5.7) | 7(3.9) |
|  | 400 - 599 | 40(26.9) | 47(27.5) |
|  | 600 - 799 | 35(23.2) | 40(23.5) |
|  | MORE THAN 800 | 66(44.2) | 77(45.1) |
| WHAT IS YOUR AVERAGE MONTHLY INCOME FROM OTHER INCOME-GENERATING ACTIVITIES? | 0 - 199 | 115(76.9) | 133(78.4) |
|  | 200 - 399 | 26(17.4) | 27(15.7) |
|  | 400 - 599 | 9(5.7) | 3(2.0) |
|  | 600- 799 | 0(0.0) | 0(0.0) |
|  | MORE THAN 800 | 0(0.0) | 3(2.0) |
|  |  |  |  |
| AVERAGE FUEL COST /PER | Before 2022 | 150(GHS350) | 170( GHS350) |
| FISHING TRIP |  |  |  |
|  | After 2022 | 150 (GHS550) | 170( GHS550) |
|  |  |  |  |
| TOP TWO PROBLEMS IN THE COMMUNITY AT THE MOMENT | High cost of living  Unemployment | 150(100.0)  75(50.0) | 170(100.0)  119(70.0) |
