# Supplementary material for: Effect of rising fuel prices on small-scale fisheries livelihoods and marine sustainability in Ghana
Source: PLoS One. 2025 Jan 13;20(1):e0317260. doi: 10.1371/journal.pone.0317260 (PMC11729924; doi:10.1371/journal.pone.0317260)
Supplement: S2 Table — (DOCX) [file pone.0317260.s002.docx]

**S2_Table. Docx**

| Variable | Value | APAM(N=150) | WINNEBA(N=170) |
| --- | --- | --- | --- |
| Purpose of catch  Catch quantity changes (over the past 5 years) | Commercial | 150(100) | 170(100) |
|  | Domestic | 0(0) | 0(0) |
|  |  |  |  |
|  | Increase | 5(3.0) | 3.4(2.0) |
|  | No change | 8(5.0) | 7(4.0) |
|  | Decrease | 138(92.0) | 160(94.0) |
| Income changes ( over the past 5 years) |  |  |  |
|  | Higher | 3(2.0) | 5(3.0) |
|  | No change | 9(6.0) | 7(4.0) |
|  | Lower | 138(92.0) | 158(93.0) |
|  |  |  |  |
| How long have you been fishing? | Less than 5 years | 6(3.8) | 10(5.9) |
|  | 5 to 10 years | 20(13.5) | 27(15.7) |
|  | 11 to 15 years | 55(36.5) | 53(31.4) |
|  | Over 15 years | 69(46.2) | 80(47.0) |
| Do you have other jobs besides fishing? | Yes  No | 9(5.7)  141(94.2) | 37(21.6)  133(78.5) |
| Has the increase in fuel prices in any way affected your business operation? | Yes | 150(100) | 167(98.1) |
|  | No | 0(0.0) | 3(1.9) |
