# Supplementary figures and images for: Effect of rising fuel prices on small-scale fisheries livelihoods and marine sustainability in Ghana
Source: PLoS One. 2025 Jan 13;20(1):e0317260. doi: 10.1371/journal.pone.0317260 (PMC11729924; doi:10.1371/journal.pone.0317260)

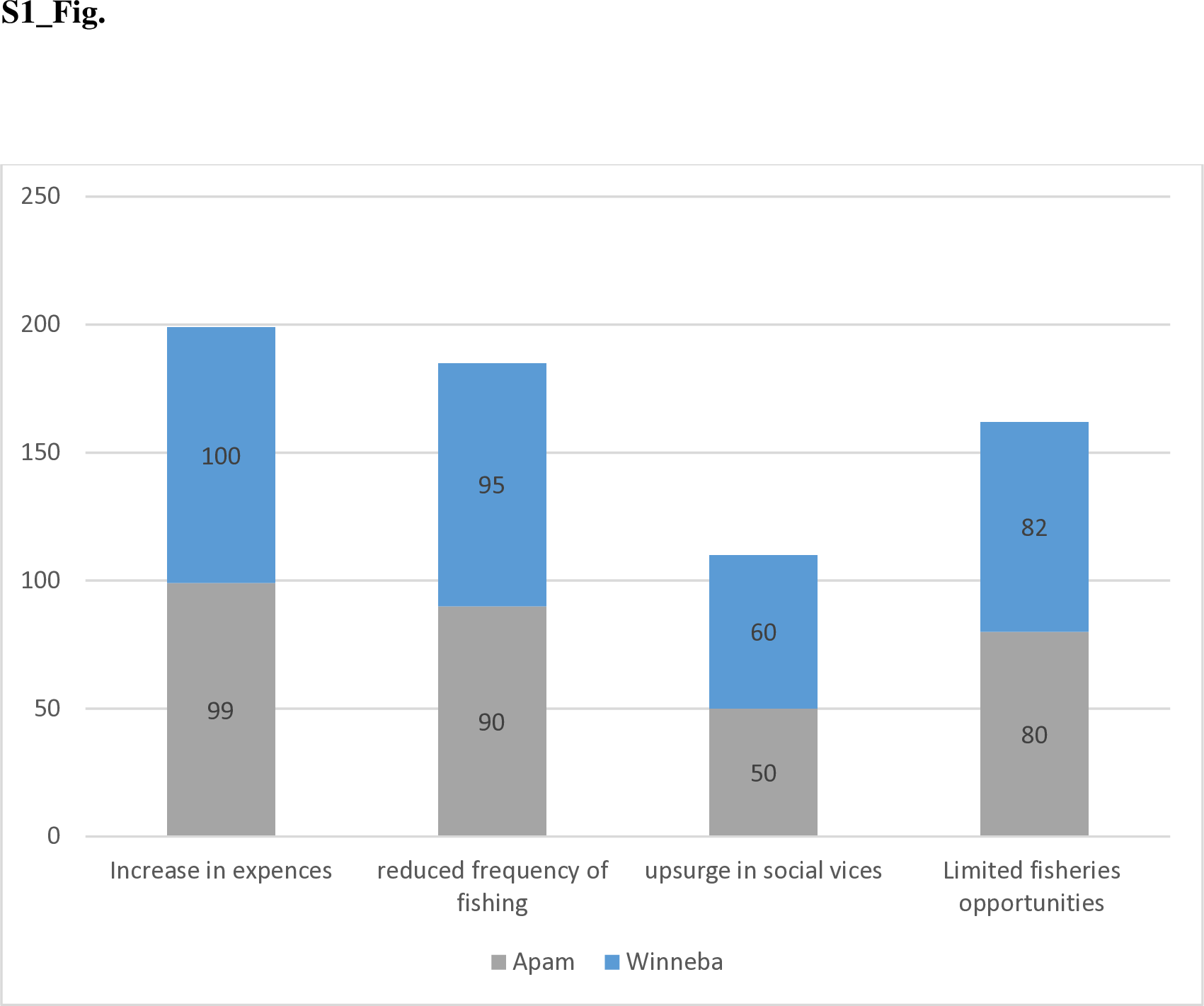

Supplement: S1 Fig — (TIF) [file pone.0317260.s003.tif]

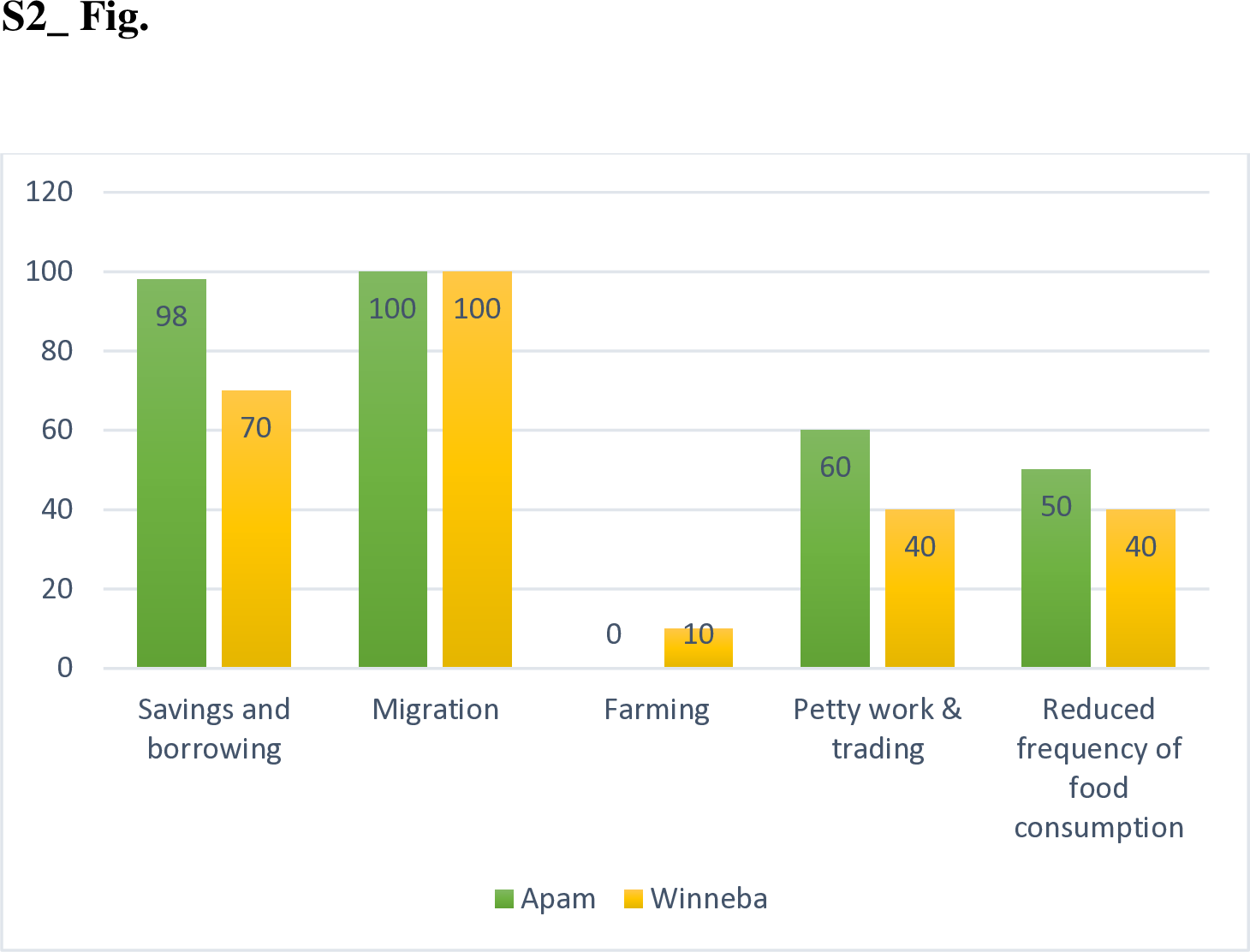

Supplement: S2 Fig — (TIF) [file pone.0317260.s004.tif]
