# Supplementary material for: Effect of rising fuel prices on small-scale fisheries livelihoods and marine sustainability in Ghana
Source: PLoS One. 2025 Jan 13;20(1):e0317260. doi: 10.1371/journal.pone.0317260 (PMC11729924; doi:10.1371/journal.pone.0317260)
Supplement: S1 File — (DOCX) [file pone.0317260.s005.docx]

**S1_ File.docx**

It has brought a lot of chaos and hardship on us. So we are appealing to the government to reduce the fuel price for us. We are unable to cater for our family as a result of the high fuel price. During the previous government there were abundance of petrol but now everything has changed. This small gallon of petrol I'm holding cost me GHS300.

( Fisher, Winneba)
