# Supplementary material for: Effect of rising fuel prices on small-scale fisheries livelihoods and marine sustainability in Ghana
Source: PLoS One. 2025 Jan 13;20(1):e0317260. doi: 10.1371/journal.pone.0317260 (PMC11729924; doi:10.1371/journal.pone.0317260)
Supplement: S2 File — (DOCX) [file pone.0317260.s006.docx]

**S2_File.docx**

The fuel price increase has impacted us negatively. Most of the petrol sellers are now engaging in black market, making the price even higher than normal. They are also sell the fuel among their committee members leaving us behind.

( Fisher and canoe owner, Apam)
