# Supplementary material for: Effect of rising fuel prices on small-scale fisheries livelihoods and marine sustainability in Ghana
Source: PLoS One. 2025 Jan 13;20(1):e0317260. doi: 10.1371/journal.pone.0317260 (PMC11729924; doi:10.1371/journal.pone.0317260)
Supplement: S3 File — (DOCX) [file pone.0317260.s007.docx]

**S3_File.docx**

The rise in the price of fuel has brought a lot of negative impacts on us and what worries me a lot is that they don't bring the fuel for those who work with it but they rather give it out to third parties as middlemen and they also double the price of the fuel for us.

(Canoe owner, Winneba)
