# Supplementary material for: Effect of rising fuel prices on small-scale fisheries livelihoods and marine sustainability in Ghana
Source: PLoS One. 2025 Jan 13;20(1):e0317260. doi: 10.1371/journal.pone.0317260 (PMC11729924; doi:10.1371/journal.pone.0317260)
Supplement: S4 File — (DOCX) [file pone.0317260.s008.docx]

**S4_File.docx**

The supply of fuel to the coast is not frequent and the increases in the prices has affected our work. We are not able to buy in large quantities for fishing operations and the catch too has declined. This has significantly reduced the number of fishing trips we embark on. So I'm appealing to the government to reduce the price of petrol and frequent supply of premix fuel for the fishermen to facilitate our work.

( Canoe owner and fisher, Winneba)
