# Supplementary material for: Effect of rising fuel prices on small-scale fisheries livelihoods and marine sustainability in Ghana
Source: PLoS One. 2025 Jan 13;20(1):e0317260. doi: 10.1371/journal.pone.0317260 (PMC11729924; doi:10.1371/journal.pone.0317260)
Supplement: S5 File — (DOCX) [file pone.0317260.s009.docx]

**S5_File.docx**

I borrow money from my canoe owner and fishmongers. Sometimes l buy food and other commodities on credit hoping to pay back during the major fishing season, if not l will starve but l have hope of paying back during the major fishing season.

( Fisher, Apam)
