# Supplementary material for: Effect of rising fuel prices on small-scale fisheries livelihoods and marine sustainability in Ghana
Source: PLoS One. 2025 Jan 13;20(1):e0317260. doi: 10.1371/journal.pone.0317260 (PMC11729924; doi:10.1371/journal.pone.0317260)
Supplement: S6 File — (DOCX) [file pone.0317260.s010.docx]

**S6_File.docx**

I depend on my small savings to survive, sometimes too l borrow money from my brother. Occasionally l work as a labourer when construction activities is going on in the community.

( Fisher , Winneba).
