# Supplementary material for: Effect of rising fuel prices on small-scale fisheries livelihoods and marine sustainability in Ghana
Source: PLoS One. 2025 Jan 13;20(1):e0317260. doi: 10.1371/journal.pone.0317260 (PMC11729924; doi:10.1371/journal.pone.0317260)
Supplement: Attachment — Submitted filename: Response to Reviewer.docx [file pone.0317260.s011.docx]

**Effect of rising fuel prices on small-scale fisheries livelihoods and marine sustainability in Ghana.**

**Academic editor**

1. Please ensure that your manuscript meets PLOS ONE's style requirements, including those for file naming. The PLOS ONE style templates can be found at

**Response**

The manuscript has been formatted in relation to PLOS ONEs style requirements.

2.Note from Emily Chenette, Editor in Chief of PLOS ONE, and Iain Hrynaszkiewicz, Director of Open Research Solutions at PLOS: Did you know that depositing data in a repository is associated with up to a 25% citation advantage (https://doi.org/10.1371/journal.pone.0230416)? If you’ve not already done so, consider depositing your raw data in a repository to ensure your work is read, appreciated and cited by the largest possible audience. You’ll also earn an Accessible Data icon on your published paper if you deposit your data in any participating repository (<https://plos.org/open-science/open-data/#accessible-data>).

**Response**

Thank you for the information

3. You indicated that ethical approval was not necessary for your study. We understand that the framework for ethical oversight requirements for studies of this type may differ depending on the setting and we would appreciate some further clarification regarding your research. Could you please provide further details on why your study is exempt from the need for approval and confirmation from your institutional review board or research ethics committee (e.g., in the form of a letter or email correspondence) that ethics review was not necessary for this study? Please include a copy of the correspondence as an ""Other"" file.

**Response**

Over the past few years l have established working relationships with fisherfolks in the Central Region of Ghana and has built trust with the communities and leadership of the fisherfolks for successful work. The already established relationship means easy entry into the communities and being affiliated with an academic community gives the team credibility to conduct ethically appropriate research with the rights and safety of the research participants and the researcher intact. Thus, the collaboration between the researchers and the case study communities in the Central Region of Ghana has developed into a long-standing respectful and reciprocal relationship.

An introductory letter has also been attached File.

5. PLOS requires an ORCID iD for the corresponding author in Editorial Manager on papers submitted after December 6th, 2016. Please ensure that you have an ORCID iD and that it is validated in Editorial Manager. To do this, go to ‘Update my Information’ (in the upper left-hand corner of the main menu), and click on the Fetch/Validate link next to the ORCID field. This will take you to the ORCID site and allow you to create a new iD or authenticate a pre-existing iD in Editorial Manager. Please see the following video for instructions on linking an ORCID iD to your Editorial Manager account: <https://www.youtube.com/watch?v=_xcclfuvtxQ>.

**RESPONSE**

ORCID iD 0009-0004-4801-0985

[0009-0004-4801-0985](https://orcid.org/0009-0004-4801-0985?lang=en)

6.Please amend your manuscript to include your abstract after the title page.

Thank you, this has been done

7.We note that Figure 4 in your submission contain [map/satellite] images which may be copyrighted. All PLOS content is published under the Creative Commons Attribution License (CC BY 4.0), which means that the manuscript, images, and Supporting Information files will be freely available online, and any third party is permitted to access, download, copy, distribute, and use these materials in any way, even commercially, with proper attribution. For these reasons, we cannot publish previously copyrighted maps or satellite images created using proprietary data, such as Google software (Google Maps, Street View, and Earth). For more information, see our copyright guidelines: http://journals.plos.org/plosone/s/licenses-and-copyright.

We require you to either (1) present written permission from the copyright holder to publish these figures specifically under the CC BY 4.0 license, or (2) remove the figures from your submission:

**RESPONSE**

Fig 4 has been removed.

8. Please review your reference list to ensure that it is complete and correct. If you have cited papers that have been retracted, please include the rationale for doing so in the manuscript text, or remove these references and replace them with relevant current references. Any changes to the reference list should be mentioned in the rebuttal letter that accompanies your revised manuscript. If you need to cite a retracted article, indicate the article’s retracted status in the References list and also include a citation and full reference for the retraction notice.

**RESPONSE**

All the necessary changes have been effected. Thank you

**Reviewer 2 Comments**

1.The abstract clearly outlines the study's objective, methodology, key findings, and implications. However, it could be more concise, focusing on the most critical points. Anyway I suggest that author must reduce redundancy, such as "The study applied a mixed-methods approach to investigate the effects of fuel price hikes on the livelihoods of coastal fisherfolk using the sustainable livelihoods framework" can be shortened. Also authors need to highlight the main findings more succinctly, possibly breaking them into bullet points for clarity

**Response**

I thank the reviewer for pointing this out. The necessary improvements have been made.

2.The introduction provides a thorough background on the issue, including relevant statistics and references. The context of global oil prices affecting local economies is well established. Also the Sustainable Livelihoods Approach (SLA) is well explained and relevant to the study. However, I think 1 and 2 should be combined as "Introduction". I think authors need to work really hard to restructure this two sections.

I also suggested that this section is dense with information. Please consider breaking it into smaller paragraphs for better readability. Also author need to clearly state the research questions earlier in the introduction to guide the reader. Please also add more recent studies to support the discussion on SLA and its application to fisheries.

**Response**

Section 1 and 2 have been combined as introduction. The section has also been broken into smaller paragraphs to make it easy to read. All the other necessary corrections has been made.

3. I think authors need to provide more details on the sampling techniques and why 220 households and 20 stakeholders were chosen. Also author need to discuss any limitations of the methods used and how they were addressed.

**Response**

The sampling techniques has been further explained see page 13 -14.

The sample size used for the study is actually 320 not 220 as initially reported by the author. It’s a typological error, kindly pardon me. The process leading to the selection of the 320 participants is explained in pages 13-15.

4. I request the authors please use more visual aids like graphs and tables to summarize key data points. Also authors must provide more direct quotes from interviews to support qualitative findings.

**Response**

Thank you for this comment, l have worked on the comments accordingly , a new chart has been added and some interview quotations have also been added.

5. Please ensure that each finding is discussed in relation to the research questions. I think authors need to highlight the practical implications of the findings for policymakers and stakeholders more explicitly. Additionally authors need to include specific recommendations for future research, and address the limitations of the study briefly and suggest how future studies could overcome these.

**Response**

Thank you for this valuable comment. The discussion section has been worked on. Kindly see the discussion section.

6.Frankly speaking, this work lacks of any interesting point and to really sure what is a really new findings from the work. Please emphasize that and explain more carefully.

**Response**

This paper contributes to the literature on global fuel price hikes and its implications for marine fisheries sustainability by investigating the effects of oil price shocks on fisheries’ livelihoods in SSF communities in Ghana. The study considers the impacts and the various coping strategies fisherfolk deploy in response to fuel price shocks. The study contributes to the literature on coastal livelihoods, ocean governance, and natural resources management. Ghana is both an oil importer and exporter; hence, examining the effects of escalating global oil prices on the local fishing industry would interest the government, development practitioners, and policymakers. The results from the study could further help to devise a more specific stimulus package tailored to cater to the unique needs of the sector involved.
