# Supplementary material for: Effect of rising fuel prices on small-scale fisheries livelihoods and marine sustainability in Ghana
Source: PLoS One. 2025 Jan 13;20(1):e0317260. doi: 10.1371/journal.pone.0317260 (PMC11729924; doi:10.1371/journal.pone.0317260)
Supplement: Attachment — Submitted filename: PONE-D-23-38320_R1_reviewer By WUBSHET.docx [file pone.0317260.s012.docx]

**PLOS ONE**

### Effect of rising fuel prices on small-scale fisheries livelihoods and marine sustainability in Ghana.

--Manuscript Draft--

| **Manuscript Number:** | PONE-D-23-38320R1 |
| --- | --- |
| **Article Type:** | Research Article |
| **Full Title:** | Effect of rising fuel prices on small-scale fisheries livelihoods and marine sustainability in Ghana. |
| **Short Title:** | Fuel Price Shocks and Coastal livelihoods |
| **Corresponding Author:** | Victor Owusu  University of Education Winneba Winneba, WINNEBA GHANA |
| **Keywords:** | Fuel-price , fisheries, sustainability, livelihoods, Ghana. |
| **Abstract:** | This study investigates the effects of fuel price hikes on the livelihoods of small-scale coastal fisherfolk in Ghana. The study applied a mixed-methods approach consisting of a questionnaire survey of 320 fisherfolk and 20 interviews with stakeholders in the fisheries sector. Increase in expenses, reduced frequency of fishing, an upsurge in social vices, and declining small-scale fisheries opportunities were found to be the main effects of fuel price hikes on fisherfolk livelihoods. The results reveal that fisherfolk experienced financial, emotional and psychological shocks due to the high cost of fuel. Dependency on savings, borrowing, petty trading, migration and farming were found to the main coping strategies. However, the various livelihood coping strategies deployed by fisherfolk were not sufficient to ameliorate their economic hardship. The findings show that fuel price hikes can contribute to reduction in fishing pressure and overcapacity despite the current socioeconomic hardship experienced by fishing households. The study recommends interest-free loans to support fisherfolk who are already engaged in small businesses. The provision of supplementary livelihoods could also improve fisherfolk’s income and well-being. |
| **Order of Authors:** | Victor Owusu, PHD |
| **Response to Reviewers:** | Academic editor  1. Please ensure that your manuscript meets PLOS ONE's style requirements, including those for file naming. The PLOS ONE style templates can be found at Response  The manuscript has been formatted in relation to PLOS ONEs style requirements. 2.Note from Emily Chenette, Editor in Chief of PLOS ONE, and Iain Hrynaszkiewicz, Director of Open Research Solutions at PLOS: Did you know that depositing data in a repository is associated with up to a 25% citation advantage (https://doi.org/10.1371/journal.pone.0230416)? If you’ve not already done so, consider depositing your raw data in a repository to ensure your work is read, appreciated and cited by the largest possible audience. You’ll also earn an Accessible Data icon on your published paper if you deposit your data in any participating repository (https://plos.org/open-science/open-data/#accessible-data).  Response  Thank you for the information  3. You indicated that ethical approval was not necessary for your study. We understand that the framework for ethical oversight requirements for studies of this type may differ depending on the setting and we would appreciate some further clarification regarding your research. Could you please provide further details on why your study is exempt from the need for approval and confirmation from your institutional review board or research ethics committee (e.g., in the form of a letter or email correspondence) that ethics review was not necessary for this study? Please include a copy of the correspondence as an ""Other"" file.  Response  Over the past few years l have established working relationships with fisherfolks in the Central Region of Ghana and has built trust with the communities and leadership of the fisherfolks for successful work. The already established relationship means easy entry |

|  | into the communities and being affiliated with an academic community gives the team credibility to conduct ethically appropriate research with the rights and safety of the research participants and the researcher intact. Thus, the collaboration between the researchers and the case study communities in the Central Region of Ghana has developed into a long-standing respectful and reciprocal relationship.  An introductory letter has also been attached File.   5. PLOS requires an ORCID iD for the corresponding author in Editorial Manager on papers submitted after December 6th, 2016. Please ensure that you have an ORCID iD and that it is validated in Editorial Manager. To do this, go to ‘Update my Information’ (in the upper left-hand corner of the main menu), and click on the Fetch/Validate link next to the ORCID field. This will take you to the ORCID site and allow you to create a new iD or authenticate a pre-existing iD in Editorial Manager. Please see the following video for instructions on linking an ORCID iD to your Editorial Manager account: [https://www.youtube.com/watch?v=_xcclfuvtxQ.](http://www.youtube.com/watch?v=_xcclfuvtxQ)   RESPONSE  ORCID iD 0009-0004-4801-0985  0009-0004-4801-0985   6. Please amend your manuscript to include your abstract after the title page. Thank you, this has been done 7. We note that Figure 4 in your submission contain [map/satellite] images which may be copyrighted. All PLOS content is published under the Creative Commons Attribution License (CC BY 4.0), which means that the manuscript, images, and Supporting Information files will be freely available online, and any third party is permitted to access, download, copy, distribute, and use these materials in any way, even commercially, with proper attribution. For these reasons, we cannot publish previously copyrighted maps or satellite images created using proprietary data, such as Google software (Google Maps, Street View, and Earth). For more information, see our copyright guidelines: [http://journals.plos.org/plosone/s/licenses-and-copyright.](http://journals.plos.org/plosone/s/licenses-and-copyright)   We require you to either (1) present written permission from the copyright holder to publish these figures specifically under the CC BY 4.0 license, or (2) remove the figures from your submission:  RESPONSE  Fig 4 has been removed.   8. Please review your reference list to ensure that it is complete and correct. If you have cited papers that have been retracted, please include the rationale for doing so in the manuscript text, or remove these references and replace them with relevant current references. Any changes to the reference list should be mentioned in the rebuttal letter that accompanies your revised manuscript. If you need to cite a retracted article, indicate the article’s retracted status in the References list and also include a citation and full reference for the retraction notice.   RESPONSE  All the necessary changes have been effected. Thank you  Reviewer 2 Comments   1. The abstract clearly outlines the study's objective, methodology, key findings, and implications. However, it could be more concise, focusing on the most critical points. Anyway I suggest that author must reduce redundancy, such as "The study applied a mixed-methods approach to investigate the effects of fuel price hikes on the livelihoods of coastal fisherfolk using the sustainable livelihoods framework" can be shortened.   Also authors need to highlight the main findings more succinctly, possibly breaking them into bullet points for clarity  Response  I thank the reviewer for pointing this out. The necessary improvements have been made.   2. The introduction provides a thorough background on the issue, including relevant statistics and references. The context of global oil prices affecting local economies is well established. Also the Sustainable Livelihoods Approach (SLA) is well explained and relevant to the study. However, I think 1 and 2 should be combined as |
| --- | --- |

|  | "Introduction". I think authors need to work really hard to restructure this two sections.  I also suggested that this section is dense with information. Please consider breaking it into smaller paragraphs for better readability. Also author need to clearly state the research questions earlier in the introduction to guide the reader. Please also add more recent studies to support the discussion on SLA and its application to fisheries.  Response  Section 1 and 2 have been combined as introduction. The section has also been broken into smaller paragraphs to make it easy to read. All the other necessary corrections has been made.   3. I think authors need to provide more details on the sampling techniques and why 220 households and 20 stakeholders were chosen. Also author need to discuss any limitations of the methods used and how they were addressed.   Response  The sampling techniques has been further explained see page 13 -14.  The sample size used for the study is actually 320 not 220 as initially reported by the author. It’s a typological error, kindly pardon me. The process leading to the selection of the 320 participants is explained in pages 13-15.   4. I request the authors please use more visual aids like graphs and tables to summarize key data points. Also authors must provide more direct quotes from interviews to support qualitative findings.   Response  Thank you for this comment, l have worked on the comments accordingly , a new chart has been added and some interview quotations have also been added.   5. Please ensure that each finding is discussed in relation to the research questions. I think authors need to highlight the practical implications of the findings for policymakers and stakeholders more explicitly. Additionally authors need to include specific recommendations for future research, and address the limitations of the study briefly and suggest how future studies could overcome these.   Response  Thank you for this valuable comment. The discussion section has been worked on. Kindly see the discussion section.   6. Frankly speaking, this work lacks of any interesting point and to really sure what is a really new findings from the work. Please emphasize that and explain more carefully. Response   This paper contributes to the literature on global fuel price hikes and its implications for marine fisheries sustainability by investigating the effects of oil price shocks on fisheries’ livelihoods in SSF communities in Ghana. The study considers the impacts and the various coping strategies fisherfolk deploy in response to fuel price shocks.  The study contributes to the literature on coastal livelihoods, ocean governance, and natural resources management. Ghana is both an oil importer and exporter; hence, examining the effects of escalating global oil prices on the local fishing industry would interest the government, development practitioners, and policymakers. The results from the study could further help to devise a more specific stimulus package tailored to cater to the unique needs of the sector involved. |
| --- | --- |
| **Additional Information:** |  |
| **Question** | **Response** |
| **Financial Disclosure**  Enter a financial disclosure statement that describes the sources of funding for the work included in this submission. Review the [submission guidelines](http://journals.plos.org/plosone/s/submission-guidelines#loc-financial-disclosure-statement) for detailed requirements. View published research articles from [*PLOS ONE*](https://journals.plos.org/plosone/) for specific | The author(s) received no specific funding for this work. |

| examples.  This statement is required for submission and **will appear in the published article** if the submission is accepted. Please make sure it is accurate. |  |
| --- | --- |
| **Funded studies**  Enter a statement with the following details:   - Initials of the authors who received each award - Grant numbers awarded to each author - The full name of each funder - URL of each funder website - Did the sponsors or funders play any role in the study design, data collection and analysis, decision to publish, or preparation of the manuscript? |  |
| Did you receive funding for this work? |  |
| **Competing Interests**  Use the instructions below to enter a competing interest statement for this submission. On behalf of all authors, disclose any [competing interests](http://journals.plos.org/plosone/s/competing-interests) that could be perceived to bias this work—acknowledging all financial support and any other relevant financial or non- financial competing interests.  This statement is required for submission and **will appear in the published article** if the submission is accepted. Please make sure it is accurate and that any funding sources listed in your Funding Information later in the submission form are also declared in your Financial Disclosure statement.  View published research articles from  [*PLOS ONE*](http://journals.plos.org/plosone/) for specific examples. | The author/s declare that they have no known competing financial interests or personal relationships that could have appeared to influence the work reported in this paper |

| **NO authors have competing interests**  Enter: *The authors have declared that no competing interests exist.*  **Authors with competing interests**  Enter competing interest details beginning with this statement:  *I have read the journal's policy and the authors of this manuscript have the following competing interests: [insert competing interests here]* |  |
| --- | --- |
| * typeset |  |
| **Ethics Statement**  Enter an ethics statement for this submission. This statement is required if the study involved:   - Human participants - Human specimens or tissue - Vertebrate animals or cephalopods - Vertebrate embryos or tissues - Field research   Write "N/A" if the submission does not require an ethics statement.  General guidance is provided below. Consult the [submission guidelines](http://journals.plos.org/plosone/s/submission-guidelines#loc-guidelines-for-specific-study-types) for detailed instructions. **Make sure that all information entered here is included in the Methods section of the manuscript.** | N/A |

| **Format for specific study types**  **Human Subject Research (involving human participants and/or tissue)**   - Give the name of the institutional review board or ethics committee that approved the study - Include the approval number and/or a statement indicating approval of this research - Indicate the form of consent obtained (written/oral) or the reason that consent was not obtained (e.g. the data were analyzed anonymously)   **Animal Research (involving vertebrate animals, embryos or tissues)**   - Provide the name of the Institutional Animal Care and Use Committee (IACUC) or other relevant ethics board that reviewed the study protocol, and indicate whether they approved this research or granted a formal waiver of ethical approval - Include an approval number if one was obtained - If the study involved *non-human primates*, add *additional details* about animal welfare and steps taken to ameliorate suffering - If anesthesia, euthanasia, or any kind of animal sacrifice is part of the study, include briefly which substances and/or methods were applied   **Field Research**  Include the following details if this study involves the collection of plant, animal, or other materials from a natural setting:   - Field permit number - Name of the institution or relevant body that granted permission |  |
| --- | --- |
|  |  |
| **Data Availability**  Authors are required to make all data underlying the findings described fully available, without restriction, and from the time of publication. PLOS allows rare exceptions to address legal and ethical concerns. See the [PLOS Data Policy](http://journals.plos.org/plosone/s/data-availability) and [FAQ](http://journals.plos.org/plosone/s/data-availability#loc-faqs-for-data-policy) for detailed information. | Yes - all data are fully available without restriction |

| A Data Availability Statement describing where the data can be found is required at submission. Your answers to this question constitute the Data Availability Statement and **will be published in the article**, if accepted. |  |
| --- | --- |
| **Important:** Stating ‘data available on request from the author’ is not sufficient. If your data are only available upon request, select ‘No’ for the first question and explain your exceptional situation in the text box. |  |
| Do the authors confirm that all data underlying the findings described in their manuscript are fully available without restriction? |  |
| **Describe where the data may be found in full sentences. If you are copying our sample text, replace any instances of XXX with the appropriate details.**   - If the data are **held or will be held in a public repository**, include URLs, accession numbers or DOIs. If this information will only be available after acceptance, indicate this by ticking the box below. For example: *All XXX files are available from the XXX database (accession number(s) XXX, XXX.*). - If the data are all contained **within the manuscript and/or Supporting Information files**, enter the following: *All relevant data are within the manuscript and its Supporting Information files.* - If neither of these applies but you are able to provide **details of access elsewhere**, with or without limitations, please do so. For example:   *Data cannot be shared publicly because of [XXX]. Data are available from the XXX Institutional Data Access / Ethics Committee (contact via XXX) for researchers who meet the criteria for access to confidential data.*  *The data underlying the results presented in the study are available from (include the name of the third party* | All relevant data are within the manuscript and its supporting information files |

| *and contact information or URL).*   - This text is appropriate if the data are owned by a third party and authors do not have permission to share the data.   * typeset |  |
| --- | --- |
| Additional data availability information: |  |

Manuscript	[Click here to access/download;Manuscript;Manuscript(clean copy).docx](https://www2.cloud.editorialmanager.com/pone/download.aspx?id=37587943&guid=1b39caf4-73d2-47a1-98e7-23f246b40b35&scheme=1)

1 **Effect of rising fuel prices on small-scale fisheries livelihoods and marine sustainability in**
2 **Ghana.**
3 Victor Owusu^1*^
4 ^1^ Department of Geography Education, University of Education, Winneba, Winneba, Ghana

5

6 ***** Corresponding author
7 **E-mail:** [vowusu@uew.edu.gh](mailto:vowusu@uew.edu.gh) **(VO)**
8 ***** The author contributed solely to this work

# 9 Abstract

10 This study investigates the effects of fuel price hikes on the livelihoods of small-scale coastal
11 fisherfolk in Ghana. The study applied a mixed-methods approach consisting of a questionnaire
12 survey of 320 fisherfolk and 20 interviews with stakeholders in the fisheries sector. Increase in
13 expenses, reduced frequency of fishing, an upsurge in social vices, and declining small-scale
14 fisheries opportunities were found to be the main effects of fuel price hikes on fisherfolk
15 livelihoods. The results reveal that fisherfolk experienced financial, emotional and psychological
16 shocks due to the high cost of fuel. Dependency on savings, borrowing, petty trading, migration
17 and farming were found to the main coping strategies. However, the various livelihood coping
18 strategies deployed by fisherfolk were not sufficient to ameliorate their economic hardship. The
19 findings show that fuel price hikes can contribute to reduction in fishing pressure and overcapacity
20 despite the current socioeconomic hardship experienced by fishing households. The study
21 recommends interest-free loans to support fisherfolk who are already engaged in small businesses.
22 The provision of supplementary livelihoods could also improve fisherfolk’s income and well-
23 being.

24

25

26

# 27 1. Introduction

28 The recent increase in global oil prices remain a major challenge for developing countries
29 because of its adverse effects on economic development (Anyars & Adabor, 2023). The rise in
30 global crude oil prices has led to significant increases in fuel prices worldwide (Acheampong &
31 Menyeh, 2022). This has increased living costs and slowed economic growth (Acheampong &
32 Menyeh, 2022; Awunyo-Vitor et al., 2018; Kamasa et al., 2020). The Ghanaian economy is highly
33 vulnerable to global oil price changes because oil as a commodity plays a key role in the nation's
34 economic activities (Anyars & Adabor, 2023; Nchor et al., 2016; Oku, 2018). Over 90 % of the
35 energy utilized in the agricultural sector in Ghana is obtained from oil (Oku, 2018). Since January
36 2022, fuel prices have increased by more than 90% (Sasu, 2022; Figure 1).
37 This study investigates the effects of fuel price hikes on the livelihoods of small-scale coastal
38 fisherfolk (SSF) in Ghana. The study also explores the various coping strategies deployed by
39 fisherfolk in response to fuel price hikes. The SSF is defined as fishing and its related activities,
40 such as processing and trading, operated by individuals and families in coastal communities
41 (Owusu & Andriesse, 2022). Fisherfolk in this study refers to fishers, fish traders and processors,
42 and any other person who depends on fishing and its related activities for their livelihood ( Owusu,
43 2019). Changes in global oil prices could significantly impact agricultural productivity, including
44 fisheries because fuel price is a key driver of inflation in Ghana (Acheampong & Menyeh, 2022;
45 Anyars & Adabor, 2023). The rising fuel prices and associated increases in food prices have caused
46 deteriorating effects on household income in developing countries (FAO, 2022).
47 Across the Global South, the fisheries sub-sector of agriculture remains a major source of
48 livelihood in many coastal communities. An estimated 38.98 million people are into capture
49 fisheries, with another 20.53 million employed in aquaculture (FAO,2020). Fish provides more
50 than 3 billion people with a 20% intake of animal proteins, reaching as high as 50 % or more in
51 countries such as Bangladesh, Cambodia, the Gambia, Ghana, Indonesia, Sierra Leone, and Sri
52 Lanka (Hasselberg et al., 2020). In Ghana, the fisheries sector contributes significantly to
53 employment, income, livelihood, nutritional security, and socioeconomic development (March &
54 Failler, 2022; Hasselberg et al., 2020; Amadu et al. 2021). The fisheries sector employs about 10%
55 of the active workforce of the population as fishers, processors, boat owners, boat builders, and
56 others in subsidiary jobs. The direct workforce for the industry includes about 140,000 fishermen
57 in the four coastal regions and 4,474 persons engaged and employed in the fishing and aquaculture
58 industry (Fisheries Commission, 2022).
59 -	**Fig 1. Changes in fuel price in Ghana (January-December 2022)**
60 The increases in fuel prices have the potential to further expose many fishing-based households in
61 developing countries to various forms of vulnerability, including poverty, hunger, and food
62 insecurity. Ghana’s oil needs are mainly dependent on imported crude oil, which makes imported
63 crude oil an essential part of the economy, and as such, changing world oil prices is a concern that
64 needs to be examined (Oku, 2018). The effects of rising global oil prices and its associated
65 challenges have been analyzed mainly from the macro/national perspectives (Awunyo-Vitor et al.,
66 2018; Dalheimer et al., 2021). Studies on the impact of oil prices on various sectors of the economy
67 at the local level in Africa are limited ( Anyars & Adabor, 2023; Nchor et al., 2016).
68 The Sustainable Livelihoods Approach (SLA) is a useful analytical tool for researchers and
69 development practitioners to understand the livelihood trajectory of rural households or individuals
70 at the local level in the midst of global oil price hikes (Li et al 2023; Owusu & Andriesse, 2022;
71 Natarajan et al., 2022). The SLA examines the link between individual or household assets and the
72 activities in which households can engage with a given set of assets (Carney et al., 1999; Scoones,
73 2009; Miani et al 2023). Access to capital assets (human, social, physical, natural, and financial)
74 and activities are enhanced or hindered by the mediating policies, institutions, and processes
75 (PIPs), as well as social relations, markets, and organizations (Allison & Horemans, 2006). The
76 key components of the SLA include the vulnerability context, livelihood capital assets, PIPs,
77 livelihood strategies, and outcomes ( Serrat, 2008).
78 The vulnerability context in SLA examines the changes in the external environment and how
79 they affect or influence people's livelihoods. The SLA is applied in this study to investigate
80 artisanal fisherfolk's vulnerabilities and livelihood strategies in the context of global oil price
81 shocks. The global fuel price hikes is considered to have triggered a major shock and stress on the
82 livelihood assets of fisherfolk. Vulnerability comprises seasonality, trends, and shock (Allison &
83 Horemans, 2006; Serrat, 2008). The trends may include decreasing fish catch, increasing fish
84 prices, and rising foodstuff costs ( Allison & Horemans, 2006). Shocks may include fuel-price
85 hikes that affect the costs of fishing inputs and market prices for fishery products (Allison &
86 Horemans, 2006; Anyars & Adabor, 2023; Nchor et al., 2016). At a household level, the illness or
87 death of a relative and the theft or loss of fishing inputs and other household items are obvious
88 shocks ( Allison & Horemans, 2006).
89 Other factors that negatively affect fishing households’ income and coastal livelihoods include
90 climate change, pandemics, structural change in the economy, and offshore oil production
91 (Bennett et al., 2020; Natarajan et al., 2022; Badjeck et al., 2010; Adjei & Overa, 2019). Other
92 drivers of change, such as overfishing, which occurs mainly because of illegal fishing activities
93 involving both industrial trawlers and artisanal fisherfolk, has also partly contributed to the
94 dwindling fish catch (Owusu, 2019; Penney et al. 2017). The industrial trawlers often engage in
95 the use of unauthorized fishing gears, transshipment, and fishing in areas reserved for the SSF

96	(Nolan 2019).

##### 97 Fig 2. Analytical framework

98 The paper proposes a modified SLA based on insights from studies on rural, coastal
99 development, and energy (Figure 2). The modified SLA pays closer attention to the political
100 economy and show how structural transformations enhance or constrain households’ access to
101 capital assets to improve livelihoods and well-being (Natarajan et al., 2022; Miani et al 2023; Li
102 et al 2023). Given the macroeconomic context of Ghana, it makes sense to include the stagnated
103 economic transformation as part of a broader process in our understanding of vulnerability (Owusu
104 & Andriesse, 2022; Natarajan et al., 2022). Over the past three decades, Ghana has made moderate
105 economic gains and transitioned from low-income to lower middle-income status with a per capita
106 GNI of USD 2,369 (World Bank, 2023). However, the economic growth has not been pro-poor
107 and skewed toward the benefit of only a few people. People working in agricultural-related jobs
108 like fishing and farming remain the poorest (Ghana Statistical Service, 2014). Coastal livelihoods
109 are sustainable when fisherfolk can earn more income, improve food security, and reduce
110 vulnerability, resulting in an overall improvement in the standard of living and well-being.
111 This paper contributes to the literature on global fuel price hikes and its implications for
112 marine fisheries sustainability by investigating the effects of oil price shocks on fisheries’
113 livelihoods in SSF communities in Ghana. The study considers the impacts and the various coping
114 strategies fisherfolk deploy in response to fuel price shocks. The study contributes to the literature
115 on coastal livelihoods, ocean governance, and natural resources management. Ghana is both an oil
116 importer and exporter; hence, examining the effects of escalating global oil prices on the local
117 fishing industry would interest the government, development practitioners, and policymakers. The
118 results from the study could further help to devise a more specific stimulus package tailored to
119 cater to the unique needs of the sector involved.
120 The study addressed the following three research questions;
121 1. What have been the effects of fuel price increases on the livelihoods of coastal fisherfolk
122 2. What are the coping strategies deployed to mitigate these impacts?
123 3. What are the implications of the study results for developing coastal livelihoods and
124 sustainable fisheries management in West Africa?
125 The study shows that fisherfolk in the Central Region of Ghana are under high socioeconomic
126 vulnerability because of fuel price hikes. Fisherfolk experienced financial, emotional and
127 psychological shocks due to the high cost of fuel which affected fisheries livelihoods. Dependency
128 on savings, borrowing, petty trading, migration and farming were found to the main coping
129 strategies. However, the various livelihood coping strategies deployed by fisherfolk were not
130 sufficient to ameliorate their economic hardship. The rest of the paper is organized as follows: the
131 next section presents the study areas, and the research methodology description. After that, the
132 results and discussion are presented, highlighting the implications of findings for improving
133 sustainable coastal management, and finally, ends with the conclusion.
134 134
135 135
136 136
137 137

# 138 2. Materials and Methods

## 139 Study context

140 In 2007, Ghana discovered oil in commercial quantities, with production beginning in 2010
141 (Owusu, 2019). The major oil and gas companies operating in Ghana include- Tullow Ghana Ltd,
142 Anadarko Petroleum, KOSMOS Energy, GNPC, Eni, Hess, and Sabre Oil and Gas, collectively
143 referred to as ‘Jubilee Partners’ ( Obeng-Odoom, 2014). In 2017, the total oil revenue received by
144 the national government was USD362.58 million (GHS1,552.13 m) (Government of Ghana,
145 2019). This amount increased to USD 723.55 m (GHS3,292.20m) by 2018. By 2019, production
146 had risen to 196,089 barrels per day and is expected to increase to 500,000 by 2025 (Ministry of
147 Finance, 2019). The increase in oil production has contributed to a significant growth rate in
148 Ghana's Gross Domestic Product (GDP). In 2019, Ghana accrued oil revenue totaling USD 938
149 million, equivalent to nine percent of government revenue (Natural Resource Governance Institute,

150	2020).

151 As a net exporter of crude oil, the surge in the global oil price should be positive for Ghana’s
152 trade balance. However, the country is highly vulnerable to global oil price shocks ( Anyars &
153 Adabor, 2023). This is because it exports its crude oil and imports a significant share of petroleum
154 products, including petrol and diesel (Oku, 2018; Acheampong & Menyeh, 2022). Years of under-
155 investment and neglect have resulted in inadequate local refining, especially at the country’s main
156 oil refinery – Tema Oil Refinery. Ghana imports about 95% of oil, with local production
157 accounting for only 5% of about 80% petroleum consumption ( Acheampong & Menyeh, 2022;
158 Anyars & Adabor, 2023).The cost of importing petroleum products increases after global oil prices
159 increase. This is because refined oil serves as inputs and raw materials for domestic industries
160 (Anyars & Adabor, 2023). Fuel prices are a vital driver of inflation in Ghana (Oku, 2018). This
161 means that any hikes in global oil prices feeding through into petroleum product imports will
162 significantly escalate inflation. During periods of increasing oil prices, oil-importing countries
163 reliant on food imports for consumption will experience a surge in their food import bill (Oku,
164 2018). The global oil price shock has exposed fundamental structural weaknesses in Ghana’s
165 economy (Acheampong & Menyeh, 2022). Despite being a net exporter of crude oil, the country
166 has failed to cushion the citizenry against escalating global oil prices. As Anyars & Adabor (2023)
167 noted, increasing oil prices generates revenue for oil-exporting countries; however, it also poses a
168 potential threat of causing inflation, recession, low growth, high food prices, and low development
169 for oil-importing countries. This exposes Ghana’s economy to shocks from global oil price with
170 adverse repercussions economic development
171 The 2015 Sustainable Development Goals (SDGs) (1, 2, & 14) seek to eradicate poverty,
172 hunger, and all forms of food insecurity and malnutrition by 2030, including the sustainable
173 management of marine and coastal ecosystems. However, the challenges to ending poverty,
174 hunger, food insecurity, and malnutrition keep growing (FAO, 2022). The Russia-Ukraine conflict,
175 the high cost of living, the COVID-19 pandemic, and climate change are affecting the livelihoods
176 of people across the world (Parker & Tyedmers, 2015; Bennett et al., 2020). The COVID-19
177 pandemic exposed the fragilities in agrifood systems and the inequities driving further increases
178 in world hunger and severe food insecurity (FAO, 2022). Prior to the recent global oil price surge,
179 the covid 19 pandemic disrupted the global fisheries supply value chains, leading to higher aquatic
180 food prices of which Ghana was no exception (Owusu et al., 2022; Bennett et al., 2020; Marcusi
181 et al., 2022). About 820 million people worldwide suffer from malnutrition, and another 736
182 million live in extreme poverty, most of which are in developing countries (Dalheimer et al., 2021).
183 Ghana is experiencing a significant increasing inequality, uncertainties in fish availability, and
184 unsustainable fisheries management (Hasselberg et al., 2020). Demand for fish exceeds the
185 domestic supply, and the shortfall is covered by approximately 200,000 tons of imports (Ministry
186 of Fisheries and Aquaculture Development, 2016). The total export for 2021 was 67,786.03mt
187 (provisional) in 2021 and 37,156.70mt in 2020, a significant 30,629.33mt increase. The country
188 imported 273,382.32mt in 2021 as against 193,226.87mt in 2020, which saw an increase of
189 80,155.45mt (Fisheries Commission, 2022).
190 Ghana has a coastline of 550km along the Gulf of Guinea. The fisheries sector consists of
191 marine capture fisheries, inland fisheries, and aquaculture. The marine fisheries sector comprises
192 four subsectors: artisanal or small-scale, semi-industrial or inshore, industrial, and tuna fisheries (
193 MoFAD 2016). Marine fishing is an important traditional economic activity of the coastal
194 communities, contributing over 80 percent of the total fish catch ( Kwadzo, 2022; Ofori-Danson
195 et al.2019 ). Marine fisheries resources include small pelagic, large pelagic (Tunas), and demersal
196 species. The most important small pelagic include round sardinellas, flat sardinellas, anchovy,
197 chub, and horse mackerels, contributing 60-70% of total marine fish production (Fisheries
198 Commission, 2022). The small pelagic contributes immensely to food and nutrition security. The
199 most important demersal species include sea breams, red snappers, groupers, grunts, croakers,
200 cephalopods, and shrimps and, together with the large pelagic (Bigeye, Yellowfin, and Skipjack),
201 contribute significantly to foreign exchange earnings (Fisheries Commission, 2022). The total
202 domestic production by artisanal canoe in 2021 amounted to 223,088.61mt compared to
203 181498.81mt in 2020 (Figure 3). Overall, the marine sector recorded 393,970.01mt in 2021 against
204 297,976mt in 2020, representing a 32% increase (Fisheries Commission, 2022).
205 **Fig 3. Total marine fish production by artisanal canoe 2000-2021 (metric tons)**
206 206
207 Two coastal communities located in the Central Region were selected for this study.
208 Winneba is a coastal town in southern Ghana. It is located in the Central Region and serves as
209 the capital of the Effutu Municipal. Winneba lies on latitude 5°22'01.5"N and longitude
210 0°37'52.2"W along the Gulf of Guinea. Winneba is situated 56 km west of Accra, Ghana’s
211 capital city, and 140 km from Cape Coast (Ankrah, 2018). Winneba is a vibrant fishing area with
212 five landing sites, 4270 fishermen, and 436 canoes (MoFAD, 2016). The main fishing grounds
213 include Eyipey, Winneba, Esuakyir, Warabeba, and Akosua Village. Mackerel, kingfish, tuna,
214 sea bream, and herring are some fish species caught here ( MoFAD, 2016 ). Besides marine
215 fishing, the Muni Lagoon is an important fishing ground for most fisherfolk (Okyere et al.,
216 2023). Three finfish and eight shellfish species are found in the lagoon. The dominant species is
217 black chin tilapia, *Sarotherodon Melanotheron* (Cichlidae) (Okyere et al., 2023). The men are
218 responsible for fishing, while the women are involved in fish processing, trading, and marketing.
219 219
220 Apam is a coastal town located along the Atlantic Ocean in Ghana. It is the capital of the
221 Gomoa West District in the Central Region of Ghana. It is approximately 68 km east of the Central
222 Regional capital, Cape Coast, and about 69 km southwest of Accra (Agyemang & Amissah, 2019).

223	It lies on the coordinates 5°18'17.91"N, 0°42'34.89"W, and 5°14'47.33"N, 0°46'35.27"W.

224 According to MoFAD ( 2016), ten landing sites, 4,062 fishermen, and 298 canoes are registered
225 in the community to support the fishing industry. The main fishing season in Apam is between
226 August and December. Some of the commonly caught fish include threadfin (*Galeoides*
227 *decadactylus*) and burrito (*Brachydeuterus auritus)* (MoFAD, 2016).

## 228 Research Methodology

229 Data collection
230 The study used a mixed-method approach to investigate the effects of fuel price hikes on the
231 livelihoods of coastal fisherfolk. The empirical data consists of a questionnaire survey of 320 SSF-
232 dependent households and 20 interviews with stakeholders in the fisheries sector. The use of both
233 quantitative and qualitative approaches provided a better comprehension of the research problem
234 and facilitated cross-validation and triangulation. The surveys and interviews were conducted
235 between July and August 2022 in the communities of Winneba and Apam in the Central Region
236 of Ghana. The data was gathered in the language the respondent best understood after obtaining
237 informed consent. The languages used included English, Fante, and Twi. The questionnaire and
238 interview guide were designed in English; however, when a respondent did not understand the
239 questions, the local languages mentioned above were used to explain the questions. To ensure
240 interviewer reliability for consistent data collection, a common standard for selecting and training
241 the research assistants were set. All the research assistants had fundamentals in conducting research
242 and fluency in English, Fanti, and Twi languages. The research assistants were trained in the data
243 collection processes and had sufficient knowledge of the issues under investigation.
244 The household questionnaire covered three broad thematic areas: socioeconomic and
245 demographic information, effects of fuel price hikes, and coping mechanisms. The questionnaire
246 included closed-ended, open-ended, and multiple-choice questions. The open-ended questions
247 allowed the respondents to freely talk about their experiences and knowledge relevant to the local
248 fishing industry and other relevant issues concerning the topic under investigation. The survey
249 lasted between 50 minutes to an hour on average per person. The major themes in the interview
250 guide was the socio-demographic information on respondents, fuel prices and fishing operations,
251 and the coping strategies deployed to mitigate the effects.
252 Sampling method and sample size
253 Quantitative and qualitative sample sizes were determined using probability and non-probability
254 sampling procedures. Simple random and purposive sampling techniques were employed in
255 selecting respondents for the household survey and interviews. According to the 2016 national
256 canoe frame survey, there were 4270 fishermen in Winneba and 4062 in Apam. The number of
257 fishermen in the study communities was similar during the survey. Information on the population
258 of fishermen was sourced from the Chief fisherman to constitute the sample frame. A total of 320
259 fisherfolk were engaged in the household survey, including 170 randomly selected fishers engaged
260 in small-scale commercial fishing from Winneba and 150 from Apam. Random sampling enables
261 each individual in the population to have an equal chance of being selected (Creswell, 2014).
262 Based on information provided by the chief fishermen that showed the presence 4270 fishermen
263 in Winneba and 4062 in Apam. The sample size was therefore determined using Yamane formula:
264 264

Sn =	𝑁 1+𝑁(𝑒)^2^

265 Where:
266 Sn = sample size
267 N = population size (8,332)
268 e = margin of error
269 269

Sn =	8332

1+8332(0.05)^2^

270 270

= 8332

21.83

271	= 381.67

272	=382 approximately

273	**Table 1. Sample structure for selecting fisherfolk**

**Study areas	Population	Proportion	Sample size**

Winneba	4,270	51%	195

Apam	4,062	49%	187

##### Total 8332 100 382

274

275 The sample sizes were proportional to the population of each area, with Winneba being slightly
276 larger (Table 1). However, 320 (84 percent) of the 382 respondents provided usable information
277 for the study. The remaining 14% either did not complete the survey or declined to do so. Even
278 though not up to the required sample size, the data is still large enough to ensure statistical validity
279 for comparison between the two areas. The sizes are within a reasonable range to detect significant
280 differences or trends in the population without introducing major biases.
281 The purposive sampling technique was used to select community leaders, government
282 officials, and NGO representatives. In-depth semi-structured interviews (N=20) were conducted
283 with community leaders (n=15), government officials (n=4), and NGO officials (n=1) to gain
284 insights into the experiences of fisherfolk concerning fuel price hikes. These stakeholders were
285 considered to have the requisite knowledge and experience with fishing and other related activities
286 in the studied communities. The goal here is to acquire an in-depth understanding of the subject of
287 the study from participants viewpoint. The sample size was determined upon saturation point (see
288 Hay 2010). These sampling techniques are efficient data-gathering methods and have been used
289 by several authors to investigate fisheries livelihoods and coastal development in West Africa
290 (Penny et al., 2017; Adjei & Overå, 2019; Marquette et al., 2002).
291 Data analysis
292 SPSS Version 23.0 and Excel were used to analyze the results from the household surveys.
293 The socio-demographic characteristics of the respondents were presented in frequency tables. The
294 effects of the fuel price hike, and coping strategies were presented in tables and charts. The
295 interviews were audio-recorded with the respondents' permission and later translated verbatim.
296 The transcribed data was manually coded and organized into relevant themes based on the study's
297 objectives and components of SLA used in the study. Qualitative content analysis technique was
298 used to interpret meaning from the content of text data (Nowell et al., 2017). The author read
299 through the transcripts severally to fully comprehend responses given by research participants and
300 highlighted particular words from the text that appear to capture critical thoughts and answer the
301 research questions. Selected narratives from the in-depth interviews were then presented as direct
302 quotations to illustrate key findings. Direct field observation of the coastal environments and
303 fishing activities further provided a depth of information. Participant observation was also
304 conducted at the fish markets and landing beaches. Information related to the organization of
305 fisheries and other related activities, such as trading and processing, were recorded. These
306 observations provided insights into the social and economic environments where fishers perform
307 daily economic activities and related social functions. Secondary data was collected from
308 published and non-published reports, online newspaper articles, and internet sources to support the
309 analysis of the study.

# 310 3.Results

311 Table 2 presents an overview of selected socio-demographic features of the two study
312 communities. The study communities in Apam recorded the highest household number, with
313 33.3% of respondents indicating they have more than 10 members in their household. Apam has
314 the highest number of fishers with no formal education (43.2%). Across the study areas, the high
315 cost of living was the topmost perceived problem (Table 2). Research participants indicated that
316 the increase in fuel prices was the major cause of the high cost of living. The majority of the
317 fisherfolk in the study areas have no alternative work apart from fishing (Table 3).
318 **Table 2. Characteristics of the study sites and persons interviewed (n)=320**

| Variable | Value | APAM | WINNEBA |
| --- | --- | --- | --- |
| AGE | BELOW 20 | 3.8 | 5.9 |
|  | 20-39 YEARS | 44.2 | 43.1 |
|  | 40-59 YEARS | 38.5 | 37.3 |
|  | ABOVE 60 | 13.5 | 13.7 |
| MARITAL STATUS |  |  |  |
|  | SINGLE | 17.3 | 29.4 |
|  | MARRIED | 59.6 | 45.1 |

319 319

|  | DIVORCED | 13.5 | 13.7 |
| --- | --- | --- | --- |
|  | SEPARATED | 9.6 | 11.8 |
| EDUCATIONAL LEVEL | NO FORMAL | 43.2 | 28.8 |
|  | EDUCATION  ELEMENTARY | 37.2 | 50.0 |
|  | SECONDARY | 17.6 | 21.2 |
|  | TERTIARY | 0.0 | 0.0 |
|  | TECHNICAL TRAINING | 2.0 | 0.0 |
| TYPE OF FISHER | CAPTAIN | 25.0 | 21.6 |
|  | CREW MEMBER | 40.4 | 41.1 |
| NUMBER OF PEOPLE IN YOUR | BOAT/CANOE OWNER  LESS THAN 5 | 34.6  13.4 | 37.3  15.7 |
| HOUSEHOLD | 6 - 10 | 61.5 | 51.0 |
|  | MORE THAN 10 | 25.1 | 25.1 |
| WHAT IS YOUR AVERAGE MONTHLY | 0 - 199 | 0.0 | 0.0 |
| INCOME FROM FISHING? | 200 - 399 | 5.7 | 3.9 |
|  | 400 - 599 | 26.9 | 27.5 |
|  | 600 - 799 | 23.2 | 23.5 |
|  | MORE THAN 800 | 44.2 | 45.1 |
| WHAT IS YOUR AVERAGE MONTHLY | 0 - 199 | 76.9 | 78.4 |
| INCOME FROM OTHER INCOME- | 200 - 399 | 17.4 | 15.7 |
| GENERATING ACTIVITIES? | 400 - 599 | 5.7 | 2.0 |
|  | 600- 799 | 0.0 | 0.0 |
|  | MORE THAN 800 | 0.0 | 2.0 |
| TOP TWO PROBLEMS IN THE | High cost of living | 100.0 | 100.0 |

320 320
321 321

COMMUNITY AT THE MOMENT

Unemployment

50.0

70.0

322 Exchange rate: USD 1 = GHS 9 as of August 2022
323 323
324 **Table 3. Key survey results**

|  | Variable Value | APAM | WINNEBA |
| --- | --- | --- | --- |
|  | Commercial  Purpose of catch Domestic | 100  - | 100  - |
|  | Increase | 3.0 | 2.0 |
|  | Catch quantity changes (over the past 5 No change | 5.0 | 4.0 |
|  | years) Decrease | 92.0 | 94.0 |
|  | Higher | 2.0 | 3.0 |
|  | Income changes (over the past 5 years) No change | 6.0 | 4.0 |
|  | Lower | 92.0 | 93.0 |
|  | How long have you been fishing? Less than 5 years | 3.8 | 5.9 |
|  | 5 to 10 years | 13.5 | 15.7 |
|  | 11 to 15 years | 36.5 | 31.4 |
|  | Over 15 years | 46.2 | 47.0 |
|  | Do you have other jobs besides fishing? Yes | 5.7 | 21.6 |
|  | No | 94.2 | 78.5 |
|  | Has the increase in fuel prices in any way affected your business operation? Yes | 100 | 98.1 |
|  | No | 0.0 | 1.9 |
| 325 |  |  |  |
| 326 |  |  |  |

327 **4.1 Perceived effects of high fuel price on fisheries livelihoods**
328 Four main issues were identified concerning how increased fuel prices affected the livelihoods
329 and well-being of coastal fisherfolk. These include an increase in expenses, reduced frequency of
330 fishing, an upsurge in social vices, and declining small-scale fisheries opportunities (Figure 4).
331 **Figure 4. Perceived effects of high fuel price on fisherfolk livelihoods.**
332 332

##### 333 Table 4. Impacts of the high fuel cost on livelihood assets of fisherfolk

334

##### Capital asset Perceived influence of high cost of fuel on livelihood assets

Natural capital	Limited fishing operation and low fish stock undermines traditional livelihoods.

Human capital	Reduced fish consumption undermines nutritional and food security; frustration, anger, and anxiety affect the health and well-being of fishing households. No provision for training or capacity building for fisherfolk to undertake alternate livelihoods

Financial capital	High cost of fuel, increased cost of living, no additional sources of finances to support daily subsistence, and low savings affect investment into other economic sectors.

Physical capital	Loss of material properties due to theft, limited ownership of productive assets such as canoes and nets.

Social capital	Weakening social capital, increases social vices erodes communities' collective action, and increases social tension.

335

336 The results from the household survey, coupled with the interviews, revealed that there has been
337 a substantial increase in expenses concerning fishing operations. The Government of Ghana
338 distributes premix fuel to coastal fishing communities at a much-subsidized price. Premix fuel
339 subsidy was introduced in Ghana's small-scale fisheries in the 1990s to deal with the high cost of
340 fuel and reduce the socioeconomic burden on the poor and vulnerable fisherfolk in coastal
341 communities ( Okyere et al., 2020; Owusu & Adjei, 2021).The subsidized premix fuel, which
342 was sold at GHS10 per gallon, has been increased to GHS25 per gallon because of the rising oil
343 prices (Addo, 2023). The majority of fisherfolk reported that the increase in fuel prices has
344 negatively affected the supply of premix fuel. Fishers have to wait for several weeks and months
345 without premix fuel supply, and the only option is to buy expensive petrol for fishing activities:
346 *Since the price of fuel started increasing, the supply of premix fuel has not been frequent. The price*
347 *of the premix fuel has also increased, which has rendered most of us unable to undertake our*
348 *fishing activities, especially those who embark on fishing activities at night.*
349 (Canoe owner, Apam)
350 *The rise in the price of petrol has brought a lot of negative effects on the fishermen. Those who*
351 *sell the petrol to us don't even get some to buy for us to purchase from them. This has caused an*
352 *increase in the price of the premix fuel, because if you want to buy the fuel unless you resort to*
353 *third parties rather than the normal channel of buying it.*
354 ( Chief Fisher, Apam)
355 Fisherfolk emphasized that even though the supply of the premix fuel was irregular, the recent fuel
356 price hikes have worsened the situation. The majority of the fisherfolk reported that the increase
357 in fuel prices has reduced the frequency of fishing operations, resulting in low catch and income.
358 Interviewed canoe owners reported spending more on fuel and food for their crew members.
359 The following extracts from the interviews with fishers aptly speak to the findings presented
360 above:
361 *I have lost about GHS2000 this month. Anytime there is an increase in fuel prices, fishermen suffer*
362 *a lot. The high fuel prices have affected everything. A small gallon of petrol is sold at GHS40 while*
363 *the bigger gallon is over GHS250. Two of my canoes have been packed because of the high fuel*
364 *price. I do not have any additional work besides fishing.*
365 (Canoe owner and Fisher, Winneba)
366 *The increases in fuel price have impacted us negatively in many ways. Most of us now incur losses*
367 *as a result of the increment. We used to fish five times during the week; however, because of the*
368 *high fuel cost, it has been reduced to only two fishing trips. The quantity of fuel determines the*
369 *duration of fishing. Our canoe owner says he cannot afford more fuel because of the higher prices.*
370 (Fisher, Apam)
371 Responses generated from the interviews with local fisherfolk—fishers, canoe owners, and local
372 leaders revealed that increases in fuel prices have a negative implication on fishing households’
373 financial capital development. Fuel constitutes a substantial component of the cost of fishing;
374 globally, about 30-50% of fishing expenditure is associated with fuel (Sumaila, 2008). In
375 developing countries, fuel constitutes about 50% of fishing expenditure within small-scale
376 fisheries (Parker & Tyedmers, 2015; Sumaila, 2008).
377 The increases in fuel prices affect other fishery-related businesses, such as processing and trading.
378 Interview with some of the fishmongers and traders indicates that the increase in fuel prices has
379 affected the quantity of fish supply and the price of fish. This point was echoed by the	fish
380 processors:
381 *The increase in fuel prices has led to the high cost of fish, which has brought us economic hardship.*
382 *We are unable to buy large quantities of fish as we did previously. This has affected our income,*
383 *and we struggle to care for our families.*
384 (Fish processor and trader, Winneba)
385 *The inflow of petrol to the coast is not frequent and this has compelled many fishermen to buy fuel*
386 *from ‘middlemen’ at expensive cost. The prices of fuel has caused an increase in the prices of fish.*
387 *About 20 years ago, when the fisheries business was good, l was able to establish a small trading*
388 *shop. However, in recent times, we barely make any profit from the fishery business to support our*
389 *families and invest in other businesses. I'm appealing to the government to reduce the price of*
390 *fuel and increase the supply for the fishermen to facilitate their work.*
391 ( Fish processor and trader, Apam)
392 The fishers reported that they sell fish at a much higher price because of increases in the prices of
393 fuel:
394 *We sell fish to the women, and they complain it is expensive, but we have to pay for the high fuel*
395 *cost. The rise in the price of petrol has affected us in so many ways, and those who even sell petrol*
396 *to us do not even get some to buy for us to purchase, which has worsened the price of the premix*
397 *fuel. We used to buy a drum/gallon of premix fuel at GHS700, but now it is sold at GHS1200-1300*.
398 (Fisher, Apam)
399 Results from the household surveys and interviews show that fisherfolk are currently undergoing
400 severe economic hardship. The increased expenditure on fishing inputs such as fuel remains a
401 major financial burden on fishing households. A recent study by Koranteng (2022) found that a
402 box of fish sold at GHS 250 is now sold between GHS450 and GHS500. The increase in fuel prices
403 has significantly led to increases in inflation, with food prices being the most affected (GSS, 2022).
404 According to the Ghana Statistical Services (2022), prices of food items such as cereal (rice, corn,
405 millet, grains, wheat, sorghum), meat, fish and other seafood, milk, dairy products, eggs, and
406 cooking oil increased significantly.
407 Many fishing households reported that their children are not able to go to school because of a lack
408 of money to cater for their needs:
409 *I find it difficult to cater to my children’s education. Though my husband is working, he does not*
410 *earn much to take care of our children, not to mention housekeeping money from him for our*
411 *feeding. I mostly pay for my children’s school fees. However, because of financial problems, they*
412 *must stay home for some time before returning to school. Some of us barely eat once in a day.*
413 (Fish processor and trader, Apam).
414 Another participant remarked:
415 *Our children are suffering because there has been a frequent rise in the price of fuel. We are*
416 *suffering; the hardship is too much for us. They told us we would not pay school fees, but we were*
417 *paying more than the school fees. Some of my children have dropped out of school because of my*
418 *inability to pay their school fees. Many of us now rely on our mothers and buyers for survival. I*
419 *have lost much money as a result of the high cost of fuel, and this has compelled most of us to*
420 *resort to borrowing.*
421 Another social problem emerging from the fuel price hikes is the upsurge of social vices and
422 increased community tension. Many fishing households reported that their money, foodstuffs, and
423 other personal properties were stolen. Many respondents revealed that the upsurge in social vices
424 has led to considerable frustration, stress, and anxiety in the community:
425 *Apart from financial problems, I go through psychological stress because I get traumatized and*
426 *think a lot about how I will survive since I do not have any other work besides fish processing.*
427 *Many have lost their jobs and livelihoods, and there has been an increase in social vices in the*
428 *community.*
429 (Fisher processor and trader, Apam)
430 Another fisher opined :
431 *The economic hardship has made some youth in the community resort to illegal ways of getting*
432 *money to cater for themselves and their family. This has led to robbery in our neighborhoods.*
433 *Since this town has no jobs, we are appealing to the government to create jobs for us.*
434 The empirical findings from this study suggest that the fuel price hikes resulted in the loss of
435 income and heightened social tension due to increased social vices. This negatively impacts the
436 human and physical capital development in coastal communities. Increased anxiety and financial
437 stress affect the well-being and health of fisherfolk in coastal communities.
438 **5.2 Livelihood Coping Strategies**
439 The livelihood strategies reported by fisherfolk include dependency on savings, borrowing, petty
440 trading, and farming. 60 % of surveyed fisherfolk in Apam depended on their savings and
441 borrowing to care for their families, while 50% of fisherfolk in Winneba depended on their savings
442 and borrowing (Figure 5).
443 **Fig 5. Coping strategies (based on fishers’ multiple-choice responses, % of fishers;’ n=320).**
444 The fishers borrow money from canoe owners, friends and families, and fish traders. They also
445 sometimes buy foodstuffs on credit from fish traders, processors, and other market women. Most
446 fishers reported that they defray their debt with fish during the major fishing season:
447 *I borrow money from the fishmongers, and l always pay back with fish. Sometimes, we buy food*
448 *and other commodities on credit, hoping to repay them during the bumper harvest season.*
449 *However, a major challenge is that because we are not getting a lot of fish during major fishing*
450 *seasons, it becomes difficult to pay back our debt*. *This has resulted in many people refusing to*
451 *lend us money and food items.*
452 (Fisher, Winneba)
453 Some fisherfolk also engaged in petty trading, such as selling coconut and used clothing. Others
454 engaged in handicrafts such as mat weaving;
455 *I save from the little I get when I sell the fish and engage in other trading activities that involve*
456 *selling used clothing to help me care for my children because I am a single parent.*
457 (Fish processor and trader, Apam)
458 In interviews, some fish traders mentioned that the lack of capital to expand the business remains
459 a major challenge to sustain their operation. Moreover, the demand for items is not encouraging,
460 making it difficult to make profit.
461 Farming is practiced by just 10 % of fisherfolk in Winneba. Some of the common crops cultivated
462 are maize and cassava. In Apam, no fisherfolk reported any farming activity. Another major
463 important coping strategy reported by fisherfolk is migration. Fishers in both study communities
464 reported that they regularly travel to fish in other coastal communities:
465 465
466 *I travelled to Weija to fish, we sometimes do not get a good catch, and the fuel cost affects us, but*
467 *we do not have any job apart from fishing. We also sometimes travel to fish at Elmina and Cape*
468 *Coast to increase our catch.*
469 ( Fisher, Winneba)
470 There are no jobs in this area, the only job we have is fishing. We regularly made enquiries from
471 other coastal areas to ascertain where there are abundant catch. When we hear of abundant catch
472 in other areas , l mobilize my crew and travel to those areas to catch fish.
473 (Canoe owner , Apam)
474 Migrating to fish is an important coping/adaptation strategy among fisherfolk in Ghana
475 (Marquette et al., 2002; Owusu, 2019).
476 Other coping strategies reported include reducing the frequency and quantity of food consumption.
477 Fisherfolk reported that the reduction in the frequency and quantity of food consumed preceded
478 the fuel price hikes. However, it became more pronounced since fuel prices started increasing.
479 50% of respondents in Apam indicated that they have reduced the frequency of food consumption
480 whilst 40 % of respondents in Winneba indicated that they have also reduced the frequency of food
481 consumption.
482 5. **Discussion**
483 This paper sought to show how recent fuel price increases affect small-scale fisheries’
484 livelihoods and marine sustainability. The study also explores the various coping strategies
485 deployed by fisherfolk in response to rising fuel prices. Increases in expenses, decreased fishing
486 frequency, upsurges in social vices, and declining small-scale fisheries opportunities were found
487 to be the main effects of high fuel prices on fisheries livelihoods. Fuel price hikes have
488 negatively impacted fishing households' financial, human, and social capital assets.
489 The financial capital assets of coastal fishing communities have deteriorated because of the
490 fuel price increases. The high fuel cost has led to decreased fishing frequency resulting in low fish
491 stock, and income (Table 4). Households that entirely depend on fishing and its related activities
492 are most affected as they do not have any viable alternative sources of income (Table 3).
493 Households with fewer livelihood options are most vulnerable to external shocks and are
494 susceptible to falling back into poverty (Andriesse, 2018; Owusu & Andriesse, 2022). The
495 increases in fuel prices have also resulted in high cost of fish at the various landing sites. This has
496 brought financial burden on women, as they are unable to buy large quantities of fish for processing
497 and trading. Previous studies show that women are among the most vulnerable in coastal fishing
498 communities due to their limited ownership of fishing assets and participation in fishing work (
499 Danso-Wiredu, 2018; Adusah-Karikari, 2015). Fishers consider fishing to be an important
500 occupation that provides their primary source of income and considering that there are no viable
501 alternative income-generating activities, the high fuel prices impose harsh economic conditions
502 and undermine traditional livelihoods. The small-scale fisheries in West Africa have suffered from
503 rapidly declining marine fish landings, resulting in decreased income and severe economic
504 hardships (Yang et al., 2019; World Bank, 2016; Adusah-Karikari, 2015). The decrease in income
505 and declining small-scale fisheries opportunities has led to disruption in the education of fisherfolk
506 children. Fisherfolk are unable to pay their children school fees leading to school dropouts or
507 temporarily stopping school.
508 The findings from this study also show that there has been an upsurge in social vices. The
509 upsurge in social vices has led to high levels of frustration, anxiety, and stress among the
510 fisherfolk.. Fisherfolk are in fear and panic because of the persistent loss of personal properties.
511 As fishing communities are recovering from the fear, panic, and anxiety induced by the COVID-
512 19 pandemic on their livelihoods ( Bennett et al., 2021; Marcusi et al. 2022), the recent fuel price
513 hikes has worsened living conditions of fisherfolk. The findings from this study reinforces the need
514 to pay more attention to the psychological and mental health needs of coastal fisherfolk. As noted
515 by Owusu & Andriese (2022) village-based livelihood analysis has overly focused on developing
516 financial and social capital with little emphasis on the well-being and health component of rural
517 folks. Without good health, fisherfolk cannot perform their daily economic activities. Increased
518 financial stress and anxiety emanating from fuel price shocks could trigger other diseases that
519 further impoverish the health status of fisherfolk.
520 Despite the uncertainties that fisherfolk have to face, the increases in fuel prices can indirectly
521 contribute towards the restoration of depleted marine fish stock through a reduction in fishing
522 pressure and overcapacity. Several scholars have concluded that Ghana's fisheries resources are
523 overfished and overexploited (Atta-Mills, 2014; Finegold et al., 2010; Hasseberg et al., 2020). The
524 rise in fuel prices, coupled with the increases in the prices of subsidized premix fuel, can translate
525 into positive marine ecological gains in the long term despite the current socioeconomic hardship
526 experienced by coastal fishing communities ( Sumaila et al. 2008). .
527 The empirical findings from the study revealed that fisherfolk engage in both marine and non
528 -marine coping strategies to mitigate against the socioeconomic hardship induced by fuel price
529 hikes. The marine -based coping strategies include traveling to fish in other coastal communities.
530 The non- marine based coping strategies included farming and petty trading. However, the various
531 livelihood strategies deployed by fisherfolk were not enough to ameliorate their economic
532 hardship. Livelihood strategies are constantly being revised to cope with contemporary trends of
533 vulnerabilities (Natarajan et al., 2022). Individuals and households explore new forms of
534 livelihoods by expanding their non-fisheries-related income sources while maintaining their base
535 in fisheries (Bryceson, 2002). The findings show that fisherfolk’s alternative sources of income
536 could hardly sustain them as majority depends solely on the fisheries (Table 3).
537 Understanding how people succeed or fail in sustaining their livelihoods in the face of global
538 oil price shocks can help design policies and interventions to assist peoples' coping strategies
539 (Allison & Horemans, 2006; Anyars & Adabor, 2023). This study provides two implications for
540 SSF-related policies in coastal Ghana: The introduction of social intervention programs and
541 supplementary livelihoods programs. There should be better-coordinated management strategies
542 by the national government and concerned stakeholders to provide incentives that contribute
543 directly to improving coastal fisherfolk livelihood conditions. In this regard, some fund can be set
544 aside and deposited into local welfare funds to compensate vulnerable fishing households during
545 global oil price shocks. It is anticipated that this type of compensation package and incentives can
546 allay coastal communities’ fears and worries about persistent fuel price increases. Cash and non-
547 cash payments are increasingly used in marine resource management to protect fishery stocks and
548 compensate for income loss (Bladon et al., 2018; Correa et al., 2014). For instance, in Bangladesh
549 and the Brazilian Amazon, fishers receive food relief items, direct cash transfers, and support for
550 alternative income-generating activities (Correa et al., 2014; Islam et al., 2016). Involved
551 government agencies should demonstrate more political will to improve human and financial
552 capital development in coastal fishing communities by -improving access to education, credit, and
553 healthcare facilities. Specific programs that target training, improved access to credit, and
554 education on savings mechanisms would improve coastal communities' human and financial
555 status. The findings show some moderate number of fisherfolk are already engaged in various
556 petty trading activities	The government can therefore introduce interest-free loans to support
557 fisherfolk who are already engaged in small businesses. Providing business training opportunities
558 could also improve the management of finances. The provision of supplementary livelihoods
559 could improve fisherfolk’s income and well-being. The government must create the enabling
560 conditions -by providing the necessary facilities and logistics to train and enhance the capacity of
561 fisherfolk. The study recommends aquaculture training for coastal fishing communities. The
562 training on best practices in small-scale aquaculture will be relevant to women in the coastal
563 communities, especially those directly connected to the fishing industry, as women are not allowed
564 to go to sea in traditional Ghanaian fisheries ( Danso-Wiredu, 2018).
565 565

# 566 6. Conclusion

567 This paper investigated the effects of fuel price increases on the small-scale fishery’s livelihoods
568 and marine sustainability in coastal communities in Ghana. The paper applied the SLA as a
569 theoretical framework to analyze artisanal fisherfolk's vulnerability, capital assets, livelihood
570 strategies, and outcomes. Increase in expenses, reduced frequency of fishing, an upsurge in social
571 vices, and declining small-scale fisheries opportunities were found to be the main effects of fuel
572 price hikes on fisherfolk livelihoods. The results reveal that fisherfolk experienced financial,
573 economic, emotional and psychological shocks due to the high cost of fuel. Dependency on
574 savings, borrowing, petty trading, migration and farming were found to the main coping strategies.
575 However, the various livelihood coping strategies deployed by fisherfolk were not sufficient to
576 ameliorate their economic hardship.
577 The study recommends the government and other concerned
578 stakeholders must expedite action on renewable energy programs to diversify the country’s energy
579 mix and improve energy security. There is a need for effective policies that can mitigate global oil
580 price shocks. The Tema oil refinery could be revamped and equipped to process local crude oil to
581 meet the high demand for petroleum products. This will help reduce economic pressures from
582 global oil price shocks in the national and local communities
583 The present study focused mainly on two fishing communities in the Central Region of Ghana.
584 Thus, future studies should consider multiple case study sites of coastal areas using qualitative and
585 quantitative approaches.
586 586
587 587
588 **Acknowledgement**
589 The author wish to thank all the research assistants and research participants for making this
590 work possible.
591 591
592 592

##### 593 References

594 Acheampong, T & Menyeh 2022. Ukraine war: how Ghana is vulnerable, and what can be done.
595 The Conversation. https://theconversation.com/ukraine-war-how-ghana-is-vulnerable-
596 and-what-can-be-done-178528
597 Addo, Z, A, B. 2023. The price of premix fuel increased from GH¢10 to GH¢25 per gallon. Citi
598 News. https://citinewsroom.com/2023/01/price-of-premix-fuel-increased-from-
599 gh%C2%A210-to-gh%C2%A225-per-gallon/
600 Adjei M, & Overå R., 2019. Opposing discourses on the offshore coexistence of the petroleum
601 industry and small-scale fisheries in Ghana. Extractive Industry & Society 6, 190–197.
602 Adom D, Sekyere PA, & Yarney L. (2019). A return to the Ghanaian cultural values of closed 603	fishing season in Ghana’s artisanal marine fishing: an essential means of restoring small 604	pelagic fish stocks. Transylvanian review of systematic and ecological research 21(3), 605	95–110.

606	Adusah-Karikari, A., 2015. Black gold in Ghana: Changing livelihoods for women in

607	communities affected by oil production. The Extractive Industries and Society, 2(1), 24-

608	32.

609 Allison, E. H., & Ellis, F., 2001. The livelihoods approach and management of small-scale
610 fisheries.Marine Policy 25(6), 377–388.
611 Allison, E. H., & Horemans, B., (2006). Putting the principles of the Sustainable Livelihoods
612 Approach into fisheries development policy and practice. Marine Policy, 30(6), 757–766.
613 Amadu I, Armah FA, Aheto DW, & Adongo CA, 2021. A study on livelihood resilience in the 614	small-scale fisheries of Ghana using a structural equation modeling approach. Ocean & 615	Coastal Management, 215, 105952.

616	Andriesse, E., (2018). Persistent fishing amidst depletion, environmental and socioeconomic 617	vulnerability in Iloilo Province, the Philippines. Ocean and Coastal Management, 157(1), 618	130–137.

619	Anyars, S. I. & Adabor, I .2023. The impact of oil price changes on inflation and disaggregated

620	inflation: Insights from Ghana. Research in Globalization, 6, 100125.

621	Atta‐Mills, J., Alder, J., & Rashid Sumaila, U., 2004. The decline of a regional fishing nation:

622	the case of Ghana and West Africa. Natural Resources Forum,

623	https://doi.org/10.1111/j.0165-0203.2004.00068.x

624	Awunyo-Vitor, D., Samanhyia, S. & Bonney, E.A. (2018). Do oil prices influence economic

625	growth in Ghana? An empirical analysis Cogent Economics & Finance, 6: 1496551.

626	Bennett, N. J., Finkbeiner,E.M, Ban,N.C., Belhabib, D., Jupiter, S.D., Kittinger, J.N.,

627	Mangubhai, S., Scholtens, J., Gill, D., & Christie , P., 2020. The COVID-19 Pandemic, 628	Small-Scale Fisheries and Coastal Fishing Communities, Coastal Management, 48:4, 629	336-347, DOI: 10.1080/08920753.2020.1766937

630 Bryceson, D.F, (2002). Multiplex livelihoods in rural Africa: Recasting the terms and conditions
631 of gainful employment. The Journal of Modern African Studies, 40(1), 1-28.
632 Carney, D., Drinkwater, M., Rusinow, T., Neefjes, K., Wanmali, S., Singh, N., 1999.
633 Livelihoods approach compared. DFID. https:/[/www.rese](http://www.researchgate.net/)a[rchgate.net](http://www.researchgate.net/) › publication ›
634 313349342_LIVELIHOODS_A.
635 Creswell, J.W. (2014). Research Design: Qualitative, Quantitative, and Mixed Methods
636 Approaches. 4th ed. Thousand Oaks, California: SAGE Publications
637 Corrêa M, A, de Almeida., Kahn J. R., Carlos , Freitas, C, and E de Carvalho (2014). Perverse
638 incentives in fishery management: The case of the defeso in the Brazilian Amazon.
639 Ecological Economics, 106, 186-194.
640 Crawford B, Gonzales L, Amin D, Nyari-Hardi B, & Sarpong YA, 2016. Report on the Baseline 641	Survey of Small Pelagic Fishing Households along the Ghana Coast. The USAID/Ghana 642	Sustainable Fisheries Management Project. (Accessed May 8, 2020).
643 https:/[/www.crc.uri.edu/download/GH2014_PGM187_C](http://www.crc.uri.edu/download/GH2014_PGM187_CRC_FIN508.pdf)R[C_FIN508.pdf](http://www.crc.uri.edu/download/GH2014_PGM187_CRC_FIN508.pdf)
644 Dalheimer, B., Herwartz, H., & Lange, A , 2021. The threat of the oil market turmoils to food
645 price stability. Energy Economics, 93(2021) 105029.
646 Finegold C, Gordon A, Mills D, Curtis L,& Pulis A ., 2010. Western Region Fisheries Sector
647 Review. World Fish Center. USAID Integrated Coastal and Fisheries Governance
648 Initiative for the Western Region, Ghana, 1–84.
649 Fisheries Commission., 2022. Annual Performance Report. Ministry of Fisheries and
650 Aquaculture Development. Accra: Ministry of Fisheries and Aquaculture Development.
651 Food and Agricultural Organisation. 2022. The state of food security and nutrition in the world,
652 repurposing food, and agricultural politics to make healthy diets more affordable.
653 Food and Agricultural Organization (FAO). 2020. The State of World Fisheries and Aquaculture
654 2020. Sustainability in action. Rome. https://doi.org/10.4060/ca9229en
655 Freduah, G., Fidelman, P., & Smith, T. F. (2019). Adaptive capacity of small‐scale coastal 656	fishers to climate and non‐climate stressors in the Western region of Ghana. The 657	Geographical Journal, 185(1), 96–110.

658	Ghana Statistical Service., 2014. Ghana Living Standards Survey 6, Main

659	Re[port.htt](http://www.statsghana.gov.gh/gssmain/fileUpload/Living%20conditions/GLSS6_)p:/[/www.statsghana](http://www.statsghana.gov.gh/gssmain/fileUpload/Living%20conditions/GLSS6_).g[ov.gh/gssmain/fileUpload/Living%20conditions/GLSS6_](http://www.statsghana.gov.gh/gssmain/fileUpload/Living%20conditions/GLSS6_)

660	Main%20Report.pdf

| 661 | Ghana Statistical Services, 2020. Statistical Bulletin, Consumer Price |
| --- | --- |
| 662 | Index.https:/[/www.statsghana](http://www.statsghana.gov.gh/gssmain/fileUpload/Price%20Indices/Bulletin_).[gov.gh/gssmain/fileUpload/Price%20Indices/Bulletin_%20](http://www.statsghana.gov.gh/gssmain/fileUpload/Price%20Indices/Bulletin_) |
| 663 | CPI%20August%202022.pdf |
| 664 | GhanaWeb, (2022). Year in review: Here is the number of times fuel prices were increased in |
| 665 | 2022. [Year in Review: Here is the number of times fuel prices were increased in 2022](https://www.ghanaweb.com/GhanaHomePage/business/Year-in-Review-Here-is-the-number-of-times-fuel-prices-were-increased-in-2022-1684070) |
| 666 | [(ghanaweb.com)](https://www.ghanaweb.com/GhanaHomePage/business/Year-in-Review-Here-is-the-number-of-times-fuel-prices-were-increased-in-2022-1684070) |
| 667 | Government of Ghana, 2019. The Budget Statement of the Government of Ghana for the 2018 |
| 668 | Financial Year. (Accessed June 6, 2021). |
| 669 | [https://www.mofep.gov.gh/sites/default/files/budget-statements/2019-Budget-Statement-](https://www.mofep.gov.gh/sites/default/files/budget-statements/2019-Budget-Statement-and-Economic-Policy_.pdf) |
| 670 | [and-Economic-Policy_.pdf](https://www.mofep.gov.gh/sites/default/files/budget-statements/2019-Budget-Statement-and-Economic-Policy_.pdf) |
| 671 |  |
| 672 | Hay, I. (2010). Qualitative research methods in human geography. Oxford Univeristy Press. |
| 673 | Hasselberg A E, Aakre I, Scholtens J, Overa J, Kolding J, Bank MS, Atter A,& Kjellevold M |
| 674 | 2020. Fish for food and nutrition security in Ghana: Challenges and opportunities. Global |
| 675 | Food Security, 26, 100380. |
| 676 | Kamasa, K, Amposah, B.D., & Forson, P. 2020. Do crude oil price changes affect economic |
| 677 | welfare? Empirical evidence from Ghana. Ghana Mining Journal, 20(1), 51-58. |
| 678 | Koranteng, K, A., 2022. China’s capture of Ghana’s fishing industry threatening food security. |
| 679 | [https://www.myjoyonline.com/chinas-capture-of-ghanas-fishing-industry-threatening-](https://www.myjoyonline.com/chinas-capture-of-ghanas-fishing-industry-threatening-food-security/) |
| 680 | [food-security/.](https://www.myjoyonline.com/chinas-capture-of-ghanas-fishing-industry-threatening-food-security/) |
| 681 | Koranteng, K.A., Ofori-Danson, P.K., & Entsua-Mensah, M , 2000. Fish and fisheries of the |
| 682 | Muni lagoon in Ghana, West Africa. Biodiversity and Conservation, 9, 487-499. |
| 683 | Kwadzo M .2022. Effects of the Closed Fishing Season on the Livelihood of Fisheries Workers: |
| 684 | A Case Study of Fisheries Workers in Elmina in the Central Region of Ghana.” World |
| 685 | Journal of Agricultural Research, 10(4):102-110 |
| 686 | Lazar N, Yankson K, Blay J, Ofori-Danson P, Markwei P, Agbogah K, Bannerman P, Sotor M, |
| 687 | Yamoah KK, & Bilisini WB,2016. Rebuilding Depleted Small Pelagic Stocks in Ghana. |
| 688 | A Closed Fishing Season Proposal to the Ministry of Fisheries and Aquaculture |
| 689 | Development. The Scientific and Technical Working Group. The USAID/Ghana |
| 690 | Sustainable Fisheries Management Project (SFMP). Narragansett, RI: Coastal Resource |
| 691 | Center, Graduate School of Oceanography, University of Rhode Island. |
| 692 | March A, & Failler P, 2022. Small-scale fisheries development in Africa: Lessons learned and |
| 693 | best practices for enhancing food security and livelihoods. Marine Policy, 136, 104925. |

694	Marcusi E. D, Rafon J.K A,& Marcusi E. S, 2022. Impact of Covid 19 and closed fishing season

695	on commercial fishers of Davao Gulf, Mindanao, Philippines. Ocean and Coast

696	Management, 217, 105997.

697	Marquette C. M, Koranteng K. A, Overå R, & Bortei-Doku A. E 2002. Small-scale fisheries, 698	population dynamics, and resource use in Africa: The case of Moree, Ghana. Ambio 31, 699	324–336.

700 Miani, A.M., Dehkordi, M.K., Narges, S., Lassois, L., Rong, T., & Hossein, A (2023). Toward
701 sustainable rural livelihoods approach: Application of grounded theory in Ghazni
702 province, Afghanistan. Applied Geography, 154, 102915.
703 Ministry of Finance., 2019. Ghana's Oil Production Estimated to go up to 500,000 per day by
704 2024 https://mofep.gov.gh/press-release/2019-02-14/ghana%27s-oil-production-
705 estimated-to-go-up-to-500%2C000-Barrels-per-day-by-2024
706 Ministry of Fisheries & Aquaculture Development, 2015. Fisheries Management Plan of Ghana. 707	A National Policy for Managing the Marine Fisheries Sector 2015-2019 56. Retrieved 708	from https://mofad.gov.gh/fisheries-management-plan-of-ghana- 2015-2019.

709 Ministry of Fisheries & Aquaculture Development., 2016. Ghana marine canoe frame survey. 710	Information report no.35. Fisheries Scientific Survey Division, Fisheries Commission, 711	Ghana.

712 Munro, G., & Sumaila, U. R. (2002). The impact of subsidies upon fisheries management and
713 sustainability: the case of the North Atlantic. Fish and Fisheries, 3(4), 233-250.
714 Natarajan, N., Newsham, A., Rigg, J & Suhardiman, D, 2022. A sustainable livelihoods
715 framework for the 21st century. World Development, 155, 105898.
716 Natural Resource Governance Institute, 2020. Ghana’s oil sales. Using commodity trading data 717	for accountability. [https://resourcegovernance.org/sites/default/files/documents/ghana-](https://resourcegovernance.org/sites/default/files/documents/ghana-oil-sales-using-commodity-trading-data-for-accountability.pdf) 718	[oil-sales-using-commodity-trading-data-for-accountability.pdf](https://resourcegovernance.org/sites/default/files/documents/ghana-oil-sales-using-commodity-trading-data-for-accountability.pdf)

719	Nchor, D., Klepac, V., & Adamec, V. 2016. Effects of oil price shocks on the Ghanaian

720	economy. Acta Universitatis Agricultural et Silviculture Mendelianae Brunensis, 64(1):

721	315-324.

722	Nolan, C. 2019. Power and Access Issues in Ghana’s Coastal Fisheries: A Political Ecology of A

723	Closing Commodity Frontier. Marine Policy 108:103621

724 Nowell, L.S., J.M. Norris, D.E. White, & N.J. Moules. (2017). Thematic analysis: Striving to 725	meet the trustworthiness criteria. International Journal of Qualitative Methods 16 (1): 726	1609406917733847

727	Obeng-Odoom, F. (2014). Black gold in Ghana: crude days for fishers and farmers? Local

728	Environment, 19(3), 259–282. https://doi.org/10.1080/13549839.2013.788481

729	Ocloo D. R. (2022). Fish crisis at Tema Harbour: Only 4 of 76 commercial fishing vessels are 730	functional. Graphic Online. [https://www.graphic.com.gh/news/general-news/fish-crisis-](https://www.graphic.com.gh/news/general-news/fish-crisis-at-tema-harbour-only-4-of-76-commercial-fishing-vessels-functional.html) 731	[at-tema-harbour-only-4-of-76-commercial-fishing-vessels-functional.html](https://www.graphic.com.gh/news/general-news/fish-crisis-at-tema-harbour-only-4-of-76-commercial-fishing-vessels-functional.html)

732	Ofori-Danson, P.K., Asiedu, B., Amponsah, S.K.K., & Crawford, B. (2019). Assessment of the 733	Socioeconomic, Food Security, and Nutrition Impacts of the 2019 Canoe Fishery Closed 734	Fishing Season in Ghana. USAID/Ghana Sustainable Fisheries Management Project.

735 Narragansett, RI: Coastal Resources Center, Graduate School of Oceanography,
736 University of Rhode Island. GH2014_SCI076_CRC. 109 pp
737 Oku, E. (2018). Effect of world oil prices on Ghana's cereal prices. Master’s degree Dissertation.
738 University of Ghana.
739 Okyere, E.Y., Adu-Boahen, K., Boateng, I., Dadson, I.Y., Boanu, N. Y & Kyeremeh, S. 2023. 740	Analysis of ecological health status of the Muni Lagoon: Evidence from heavy metal 741	content in its water and fish samples. Geography and environment, 10, e00115.

742 Okyere, I., Chuku, E. O., Ekumah, B., Angnuureng, D. B., Boakye-Appiah, J. K., Mills, D. J., ... 743	& Crawford, B. 2020. Physical distancing and risk of COVID-19 in small-scale fisheries: 744	a remote sensing assessment in coastal Ghana. Scientific reports, 10(1), 1–13.

745 Owusu V & Andriesse E, 2020. From free access regime to the closed fishing season: Lessons 746	from small-scale coastal fisheries in the Western Region of Ghana. Marine Policy, 121, 747	104162.

748	Owusu V, & Andriesse E , 2022. Local differentiation and adapting to climate change in coastal

749	Ghana. Geographical Review. DOI: 10.1080/00167428.2021.2023530.

750	Owusu, V & Adjei, M., 2021. Politics, power, and unequal access to fisheries subsidies among 751	small-scale coastal fisherfolk in Ghana. Ocean and Coastal Management, 214 (2021) 752	10592.

753	Owusu, V., Tamatey, E.L, Adjei, M, Ogbe, M .2023. Impact of offshore petroleum extraction 754	and ‘ocean grabbing’ on small-scale fisheries and coastal livelihoods in Ghana. Journal of 755	Maritime Studies.22(17)

756 Owusu., V., 2019. Impacts of the petroleum industry on the livelihoods of fisherfolk in Ghana: 757	A case study of the Western Region. The Extractive Industries and Society, 6(4), 1256– 758	1264.

759 Parker, W., R., & Tyedmers, P., H. (2015). Fuel consumption of global fishing fleets: current
760 understanding and knowledge gaps. Fish and Fisheries, pp. 16, 684–696.
761 Penney, R., Wilson, G., & Rodwell, L. (2017). Managing Sino-ghanaian fishery relations: A
762 political ecology approach. Marine Policy, pp. 79, 46–53.
763 Perry, R.I., Sumaila, U.R., 2007. Marine ecosystem variability and human community responses:
764 The example of Ghana, West Africa. Mar. Policy 31, 125–134.
765 Sasu, D.D.(2023). Weekly prices of gasoline in Ghana from October 19, 2020, to April 17, 2023.
766 <https://www.statista.com/statistics/1200108/weekly-gasoline-prices-in-ghana/>
767 Scoones, I. (2009). Livelihoods perspectives and rural development. Journal of Peasant Studies,

768	36(1), 171–196.

769	Serrat, O. (2008). The sustainable livelihoods approach. Regional & Sustainable Development

770	Department, Asian Development. Manila. The Philippines.

771	Sumaila. U.R, The. L., Watson, R., Tyedmers, P., Pauly, D. 2008. Fuel price increase, subsidies, 772	overcapacity, and resource sustainability, ICES Journal of Marine Science, 65(6), 832– 773	840.

774 World Bank,2016. Safety and sustainability for small-scale fishers in West Africa.
775 https:/[/www.worldbank.or](http://www.worldbank.org/en/news/feature/2016/05/16/safety-and-sustainability-for-)g[/en/news/feature/2016/05/16/safety-and-sustainability-for-](http://www.worldbank.org/en/news/feature/2016/05/16/safety-and-sustainability-for-)
776 small-scale-fishers-in-west-africa.
777 World Bank.2023 World Bank Country and Lending
778 Groups.https://datahelpdesk.worldbank.org/knowledgebase/articles/906519
779 Yang, J., Owusu, V., Andriesse, E., & Dziwornu Ablo, A. 2019. In-situ adaptation and coastal 780	vulnerabilities in Ghana and Tanzania. The Journal of Environment & Development, 781	28(3), 282-308.

782

Figure	[Click here to access/download;Figure;Fig4.docx](https://www2.cloud.editorialmanager.com/pone/download.aspx?id=37543573&guid=eaaba8c3-3d6e-43b9-97d3-82a72abd732a&scheme=1)

250

200

150

100

50

0

Increase in expences

reduced frequency of

fishing

upsurge in social vices

Limited fisheries

opportunities

Apam	Winneba

**Fig.4 Perceived effects of high fuel price on fisherfolk livelihoods ( (based on fishers’ multiple-choice responses, % of fishers;’ n=320).**

Figure	[Click here to access/download;Figure;Fig5.docx](https://www2.cloud.editorialmanager.com/pone/download.aspx?id=37543580&guid=562ee856-6623-470e-9b00-a3936ecd4bdd&scheme=1)

120

100

80

60

40

20

0

Savings and

borrowing

Migration

Farming	Petty work &	Reduced

trading	frequency of food

consumption

Apam	Winneba

###### Figure 5. Coping strategies (based on fishers’ multiple-choice responses, % of fishers;’ n=320).

Figure	[Click here to access/download;Figure;Fig 1.docx](https://www2.cloud.editorialmanager.com/pone/download.aspx?id=37543568&guid=73ff7d88-64a0-40a0-a562-5ceb23f91fde&scheme=1)

**Price in Ghanaian cedis per liter**

16.82

13.20

12.63

11.55

11.00

10.10	10.30

9.39

8.29	8.50

7.42

6.50

Fig 1. Changes in fuel price in Ghana (January-December 2022) Source (GhanaWeb, 2022)

**18.00**

**16.00**

**14.00**

**12.00**

**10.00**

**8.00**

**6.00**

**4.00**

**2.00**

**0.00**

**Month**

Exchange rate: USD 1 = GHS 9 as of August 2022

Figure	[Click here to access/download;Figure;Fig 2.docx](https://www2.cloud.editorialmanager.com/pone/download.aspx?id=37587957&guid=330dbb1f-0e75-4db7-b67f-fe16f8128ccb&scheme=1)

##### Fig. 2 Analytical framework

Source: Adapted from (Carney et al., 1999)

Figure	[Click here to access/download;Figure;Fig 3.docx](https://www2.cloud.editorialmanager.com/pone/download.aspx?id=37587958&guid=eaea3635-108f-4bc4-9acc-cc5bec849052&scheme=1)

300000

250000

200000

150000

Catch (MT)

100000

50000

0

2000	2002	2004	2006	2008	2010	2012	2014	2016	2018	2020	2021

Fig 3*.* Total marine fish production by artisanal canoe 2000-2021 (metric tons)

Source (Fisheries Commission, 2021 & 2022)

Supporting Information

[Click here to access/download](https://www2.cloud.editorialmanager.com/pone/download.aspx?id=37587959&guid=cabd31fc-73e8-4599-a76c-76fbf7b62dec&scheme=1)

[**Supporting Information** Supporting information files.docx](https://www2.cloud.editorialmanager.com/pone/download.aspx?id=37587959&guid=cabd31fc-73e8-4599-a76c-76fbf7b62dec&scheme=1)

Revised Manuscript with Track Changes

1

2

3

4

5

6 Effect of rising fuel prices on small-scale fisheries livelihoods and marine sustainability in
7 **Ghana.**

**Formatted:** No Spacing, Left, Line spacing: single

8

9

10	Victor Owusu^1*^

11

12	^1^ Department of Geography Education, University of Education, Winneba, Winneba, Ghana

13

14

15

16 ***** Corresponding author
17 **E-mail:** [vowusu@uew.edu.gh](mailto:vowusu@uew.edu.gh) **(VO)**
18 ***** The author contributed solely to this work

19

**Formatted:** Font: 18 pt

20

21

22

23

24

**Formatted:** Tab stops: 1.14", Left

**Formatted:** Indent: First line: 0.3"

25

26	**Abstract**

27 This study investigates the effects of fuel price hikes on the livelihoods of small-scale coastal
28 fisherfolk in Ghana. The study applied a mixed-methods approach ~~to investigate the effects of fuel~~
29 ~~price hikes on the livelihoods of coastal fisherfolk using the sustainable livelihoods framework.~~
30 ~~The empirical data~~ consisting of a questionnaire survey of 3~~2~~20 fisherfolk and 20 interviews with
31 stakeholders in the fisheries sector. Increase in expenses, reduced frequency of fishing, an upsurge
32 in social vices, and declining small-scale fisheries opportunities were found to be the main effects
33 of fuel price hikes on fisherfolk livelihoods. The results reveal that fisherfolk experienced
34 financial~~economic~~, emotional and psychological shocks due to the high cost of ~~fuel which affected~~
35 ~~fisheries and other fish value chain services~~.fuel. Dependency on savings, borrowing, petty trading,
36 migration and farming were found to the main coping strategies. However, ~~The~~the various
37 livelihood coping strategies~~strategies~~ deployed by fisherfolk were not sufficient to ameliorate their
38 economic hardship. ~~Fishing in coastal lagoons and other inland water bodies as a coping strategy~~
39 ~~during the closed fishing season is counterproductive to the rebuilding of depleted fish stock.~~ The
40 findings show that fuel price hikes can contribute to reduction in fishing pressure and overcapacity
41 despite the current socioeconomic hardship experienced by fishing households. The study
42 recommends interest-free loans to support fisherfolk who are already engaged in small businesses.
43 The provision of supplementary livelihoods could also improve fisherfolk’s income and well-
44 being.~~government and other concerned stakeholders should provide incentives that contribute~~
45 ~~directly to improving the standard of living of fishing households~~.

46

47

48

49

50

51

52

53

54

55

56

57

58	**1. Introduction**

**Formatted:** Font: 18 pt

59 The recent increase in global oil prices remain a major challenge for developing countries

**Formatted:** Indent: First line: 0.3"

60 because of its adverse effects on economic development (Anyars & Adabor, 2023). The rise in
61 global crude oil prices has led to significant increases in fuel prices worldwide (Acheampong &
62 Menyeh, 2022). This has increased living costs and slowed economic growth (Acheampong &
63 Menyeh, 2022; Awunyo-Vitor et al., 2018; Kamasa et al., 2020). The Ghanaian economy is highly
64 vulnerable to global oil price changes because oil as a commodity plays a key role in the nation's
65 economic activities (Anyars & Adabor, 2023; Nchor et al., 2016; Oku, 2018). Over 90 % of the
66 energy utilized in the agricultural sector in Ghana is obtained from oil (Oku, 2018). Since January
67 2022, fuel prices have increased by more than 90% (Sasu, 2022; Figure 1).
68 This study investigates the effects of fuel price hikes on the livelihoods of small-scale coastal
69 fisherfolk (SSF) in Ghana. The study also explores the various coping strategies deployed by
70 fisherfolk in response to fuel price hikes. The SSF is defined as fishing and its related activities,
71 such as processing and trading, operated by individuals and families in coastal communities
72 (Owusu & Andriesse, 2022). Fisherfolk in this study refers to fishers, fish traders and processors,
73 and any other person who depends on fishing and its related activities for their livelihood ( Owusu,
74 2019). Changes in global oil prices could significantly impact agricultural productivity, including
75 fisheries because fuel price is a key driver of inflation in Ghana (Acheampong & Menyeh, 2022;
76 Anyars & Adabor, 2023). The rising fuel prices and associated increases in food prices have caused
77 deteriorating effects on household income in developing countries (FAO, 2022).
78 ~~This study investigates the effects of fuel price hikes on the livelihoods of small-scale coasta~~l
79 ~~fisherfolk (SSF) in Ghana. SSF is defined as fishing and its related activities, such as processing~~
80 ~~and trading, operated by individuals and families in coastal communities (Owusu & Andriesse~~,
81 ~~2022). Fisherfolk in this study refers to fishers, fish traders and processors, and any other person~~
82 ~~who depends on fishing and its related activities for their livelihood~~.
83 Across the Global South, the fisheries sub-sector of agriculture remains a major source of
84 livelihood in many coastal communities. An estimated 38.98 million people are into capture
85 fisheries, with another 20.53 million employed in aquaculture (FAO,2020). Fish provides more
86 than 3 billion people with a 20% intake of animal proteins, reaching as high as 50 % or more in
87 countries such as Bangladesh, Cambodia, the Gambia, Ghana, Indonesia, Sierra Leone, and Sri
88 Lanka (Hasselberg et al., 2020). In Ghana, the fisheries sector contributes significantly to
89 employment, income, livelihood, nutritional security, and socioeconomic development (March &
90 Failler, 2022; Hasselberg et al., 2020; Amadu et al. 2021). The fisheries sector employs about 10%
91 of the active workforce of the population as fishers, processors, boat owners, boat builders, and
92 others in subsidiary jobs. The direct workforce for the industry includes about 140,000 fishermen
93 in the four coastal regions and 4,474 persons engaged and employed in the fishing and aquaculture
94 industry (Fisheries Commission, 2022).
95 ~~-~~	Fig 1. Changes in fuel price in Ghana (January-December 2022)

96	-

**Formatted:** List Paragraph, Justified, Line spacing: 1.5 lines, Bulleted + Level: 1 + Aligned at: 0.25" + Indent at: 0.5"

**Formatted:** Font: (Default) Times New Roman

97 The increases in fuel prices have the potential to further expose many fishing-based households in
98 developing countries to various forms of vulnerability, including poverty, hunger, and food
99 insecurity. Ghana’s oil needs are mainly dependent on imported crude oil, which makes imported
100 crude oil an essential part of the economy, and as such, changing world oil prices is a concern that
101 needs to be examined (Oku, 2018). The effects of rising global oil prices and its~~their~~ associated
102 challenges have been analyzed mainly from the macro/national perspectives (Awunyo-Vitor et al.,
103 2018; Dalheimer et al., 2021). Studies on the impact of oil prices on various sectors of the economy
104 ~~in~~ at the local level in Africa are limited ( Anyars & Adabor, 2023; Nchor et al., 2016).

**Formatted:** Font color: Auto

106 ~~This paper contributes to the literature on global fuel price hikes and its implications for marine~~
107 ~~fisheries sustainability by investigating the effects of oil price shocks on fisheries’ livelihoods in~~
108 ~~SSF communities in Ghana. The study considers the impacts and the various coping strategies~~
109 ~~fisherfolk deploy in response to fuel price shocks. The study contributes to the literature on coastal~~
110 ~~livelihoods, ocean governance, and natural resources management. Ghana is both an oil importer~~
111 ~~and exporter; hence, examining the effects of escalating global oil prices on the local fishing~~
112 ~~industry would interest the government, development practitioners, and policymakers. The results~~
113 ~~from the study could further help to devise a more specific stimulus package tailored to cater to~~
114 ~~the unique needs of the sector involved~~.
115 ~~The study addressed the following three research questions~~;
116 ~~1.	What have been the effects of fuel price increases on the livelihoods of coastal fisherfolk~~

**Formatted:** Font: (Default) Times New Roman, 12 pt

117 ~~2.	What are the coping strategies deployed to mitigate these impacts?~~

**Formatted:** Normal, No bullets or numbering

118 ~~3.	What are the implications of the study results for developing coastal livelihoods and~~

**Formatted:** Font: (Default) Times New Roman, 12 pt, Font color: Black

119 ~~sustainable fisheries management in West Africa?~~
120 ~~The empirical data gathered comprised 220 household surveys and 20 in-depth interviews with~~
121 ~~stakeholders at the local district, regional, and national levels. The rest of the paper is organized~~
122 ~~as follows: the next section presents the study's theoretical framework, the study areas, and the~~
123 ~~research methodology description. After that, the results and discussion are presented, highlighting~~
124 ~~the implications of findings for improving sustainable coastal management, and finally, ends with~~
125 ~~the conclusion.~~
126 ~~2. Sustainable small-scale fisheries livelihoods~~
127 The Sustainable Livelihoods Approach (SLA) is a useful analytical tool for researchers and

**Formatted:** Indent: First line: 0.3"

128 development practitioners to understand the livelihood trajectory of rural households or individuals
129 at the local level in the midst of global oil price hikes (Li et al 2023~~Allison & Horemans, 2006~~;
130 Owusu & Andriesse, 2022; Natarajan et al., 2022). The SLA examines the link between individual
131 or household assets and the activities in which households can engage with a given set of assets
132 (Carney et al., 1999; Scoones, 2009; Miani et al 2023). Access to capital assets (human, social,
133 physical, natural, and financial) and activities are enhanced or hindered by the mediating policies,
134 institutions, and processes (PIPs), as well as social relations, markets, and organizations (Allison
135 & Horemans, 2006). The key components of the SLA include the vulnerability context, livelihood
136 capital assets, PIPs, livelihood strategies, and outcomes ( Serrat, 2008)..
137 ~~The ocean is a natural capital from which fish and other resources are extracted. Other forms~~
138 ~~of natural capital available for coastal dwellers may include land. The available sources of money,~~
139 ~~such as savings, credit, insurance, and remittances, make up the financial capital. Human capital~~
140 ~~includes the knowledge, capability to work, health, education, ability, and skills required for~~
141 ~~fishing and its related activities. Regarding social capital, fisherfolk have networks and~~
142 ~~connections, associations, and membership organizations that provide avenues for collective~~
143 ~~action. The physical capital available at the household level may include houses and fishing inputs~~
144 ~~such as nets and canoes. At the community level, available physical assets may include roads~~,
145 ~~landing beaches, harbors, schools, and health centers~~.
146 The vulnerability context in SLA examines the changes in the external environment and how
147 they affect or influence people's livelihoods. The SLA is applied in this study to investigate
148 artisanal fisherfolk's vulnerabilities and livelihood strategies in the context of global oil price
149 shocks. .
150 T~~Within this study, t~~he global fuel price hikes~~rising fuel cost~~ is considered to have triggered
151 a major shock and stress on the livelihood assets of fisherfolk. Vulnerability comprises seasonality,
152 trends, and shock (Allison & Horemans, 2006; Serrat, 2008). The trends may include decreasing
153 fish catch, increasing fish prices, and rising foodstuff costs ( Allison & Horemans, 2006).. Shocks
154 may include fuel-price hikes that affect the costs of fishing inputs and market prices for fishery
155 products (Allison & Horemans, 2006; Anyars & Adabor, 2023; Nchor et al., 2016). At a household
156 level, the illness or death of a relative and the theft or loss of fishing inputs and other household
157 items are obvious shocks ( Allison & Horemans, 2006).

**Formatted:** Indent: First line: 0.3"

158 Other factors that negatively affect fishing households’ income and coastal livelihoods include
159 climate change, pandemics, structural change in the economy, and offshore oil production
160 (Bennett et al., 2020; Natarajan et al., 2022; Badjeck et al., 2010; Adjei & Overa, 2019). Other
161 drivers of change, such as overfishing, which occurs mainly because of illegal fishing activities
162 involving both industrial trawlers and artisanal fisherfolk, has also partly contributed to the
163 dwindling fish catch (Owusu, 2019; Penney et al. 2017). The industrial trawlers often engage in
164 the use of unauthorized fishing gears, transshipment, and fishing in areas reserved for the SSF

165	(Nolan 2019).

166 Fig 2. Analytical framework
167 The paper proposes a modified SLA based on insights from studies on rural, coastal
168 development, and energy (Figure 2~~1~~). The modified SLA pays closer attention to the political
169 economy and show how structural transformations enhance or constrain households’ access to
170 capital assets to improve livelihoods and well-being (Natarajan et al., 2022; Miani et al 2023; Li
171 et al 2023). Given the macroeconomic context of Ghana, it makes sense to include the stagnated
172 economic transformation as part of a broader process in our understanding of vulnerability (Owusu
173 & Andriesse, 2022; Natarajan et al., 2022). Over the past three decades, Ghana has made moderate
174 economic gains and transitioned from low-income to lower middle-income status with a per capita
175 GNI of USD 2,369 (World Bank, 2023). However, the economic growth has not been pro-poor
176 and skewed toward the benefit of only a few people. People working in agricultural-related jobs
177 like fishing and farming remain the poorest (Ghana Statistical Service, 2014). ~~See also section 3~~.
178 Coastal livelihoods are sustainable when fisherfolk can earn more income, improve food security,
179 and reduce vulnerability, resulting in an overall improvement in the standard of living and well-
180 being.
181 ~~Fisherfolk utilize their available capital assets to construct their livelihood in the face of~~
182 ~~multiple vulnerabilities. In-situ livelihood strategies include income diversification activities such~~
183 ~~as aquaculture, small business, tourism, farming, craft work, and other jobs in construction and~~
184 ~~factories in the local community (Yang et al., 2019; Owusu, 2019; Perry & Sumaila, 2007;~~
185 ~~Marquette et al., 2002). Diversification of livelihood activities has become an integral component~~
186 ~~of rural households /individuals (Natarajan et al., 2022). Migration as a coping strategy is a vita~~l
187 ~~component of many fishing household livelihood activities where the men migrate for fishing and~~
188 ~~women travel to work in the post-harvest processing and marketing activities ( Marquette et al.~~,
189 ~~2002; Allison & Horemans, 2006; Freduah et al., 2019; Owusu, 2019). Coastal livelihoods are~~
190 ~~sustainable when fisherfolk can earn more income, improve food security, and reduce~~
191 ~~vulnerability, resulting in an overall improvement in the standard of living and well-being.~~This
192 paper contributes to the literature on global fuel price hikes and its implications for marine fisheries
193 sustainability by investigating the effects of oil price shocks on fisheries’ livelihoods in SSF
194 communities in Ghana. The study considers the impacts and the various coping strategies
195 fisherfolk deploy in response to fuel price shocks. The study contributes to the literature on coastal
196 livelihoods, ocean governance, and natural resources management. Ghana is both an oil importer
197 and exporter; hence, examining the effects of escalating global oil prices on the local fishing
198 industry would interest the government, development practitioners, and policymakers. The results
199 from the study could further help to devise a more specific stimulus package tailored to cater to
200 the unique needs of the sector involved.

**Formatted:** Font: Font color: Auto

201 The study addressed the following three research questions;
202 1. What have been the effects of fuel price increases on the livelihoods of coastal fisherfolk

**Formatted:** Numbered + Level: 1 + Numbering Style: 1, 2, 3, … + Start at: 1 + Alignment: Left + Aligned at: 0.25" + Indent at: 0.5"

203 2. What are the coping strategies deployed to mitigate these impacts?
204 3. What are the implications of the study results for developing coastal livelihoods and
205 sustainable fisheries management in West Africa?
206 The study shows that fisherfolk in the Central Region of Ghana are under high socioeconomic

**Formatted:** Font: (Default) Times New Roman, 12 pt

207 vulnerability because of fuel price hikes. Fisherfolk experienced financial, emotional and

**Formatted:** Font: (Default) Times New Roman, 12 pt

208 psychological shocks due to the high cost of fuel which affected fisheries livelihoods. Dependency

**Formatted:** Font: (Default) Times New Roman, 12 pt

209 on savings, borrowing, petty trading, migration and farming were found to the main coping

**Formatted:** Font: (Default) Times New Roman, 12 pt

210 strategies. However, the various livelihood coping strategies deployed by fisherfolk were not

**Formatted:** Font: (Default) Times New Roman, 12 pt

211 sufficient to ameliorate their economic hardship. The rest of the paper is organized as follows: the
212 next section presents the study areas, and the research methodology description. After that, the
213 results and discussion are presented, highlighting the implications of findings for improving
214 sustainable coastal management, and finally, ends with the conclusion.

#### 219 2~~3~~. Materials and Methods

**Formatted:** Font: 18 pt

220	**Study context**

**Formatted:** Font: 16 pt

221 In 2007, Ghana discovered oil in commercial quantities, with production beginning in 2010

**Formatted:** Indent: First line: 0.3"

222 (Owusu, 2019). The major oil and gas companies operating in Ghana include- Tullow Ghana Ltd,
223 Anadarko Petroleum, KOSMOS Energy, GNPC, Eni, Hess, and Sabre Oil and Gas, collectively
224 referred to as ‘Jubilee Partners’ ( Obeng-Odoom, 2014). In 2017, the total oil revenue received by
225 the national government was USD362.58 million (GHS1,552.13 m) (Government of Ghana,
226 2019). This amount increased to USD 723.55 m (GHS3,292.20m) by 2018. By 2019, production
227 had risen to 196,089 barrels per day and is expected to increase to 500,000 by 2025 (Ministry of
228 Finance, 2019). The increase in oil production has contributed to a significant growth rate in
229 Ghana's Gross Domestic Product (GDP). In 2019, Ghana accrued oil revenue totaling USD 938
230 million, equivalent to nine percent of government revenue (Natural Resource Governance Institute,

231	2020).

232 As a net exporter of crude oil, the surge in the global oil price should be positive for Ghana’s
233 trade balance. However, the country is highly vulnerable to global oil price shocks ( Anyars &
234 Adabor, 2023). This is because it exports its crude oil and imports a significant share of petroleum
235 products, including petrol and diesel (Oku, 2018; Acheampong & Menyeh, 2022). Years of under-
236 investment and neglect have resulted in inadequate local refining, especially at the country’s main
237 oil refinery – Tema Oil Refinery. ~~The country imports about 80% of finished petroleum products~~
238 ~~(Acheampong & Menyeh, 2022)~~.Ghana imports about 95% of oil, with local production
239 accounting for only 5% of about 80% petroleum consumption ( Acheampong & Menyeh, 2022;
240 Anyars & Adabor, 2023). The cost of importing petroleum products increases after global oil
241 prices increase. This is because refined oil serves as inputs and raw materials for domestic
242 industries (Anyars & Adabor, 2023). Fuel prices are a vital driver of inflation in Ghana (Oku,
243 2018). This means that any hikes in global oil prices feeding through into petroleum product
244 imports will significantly escalate inflation. During periods of increasing oil prices, oil-importing
245 countries reliant on food imports for consumption will experience a surge in their food import bill
246 (Oku, 2018). The global oil price shock has exposed fundamental structural weaknesses in Ghana’s

**Formatted:** Not Highlight

247 economy (Acheampong & Menyeh, 2022). Despite being a net exporter of crude oil, the country
248 has failed to cushion the citizenry against escalating global oil prices. ~~This has adversely affected~~
249 ~~the livelihoods and economic condition of coastal fishing communities~~. As Anyars & Adabor
250 (2023) noted, increasing oil prices generates revenue for oil-exporting countries; however, it also
251 poses a potential threat of causing inflation, recession, low growth, high food prices, and low
252 development for oil-importing countries. ~~Ghana imports about 95 % of oil, with local production~~

**Formatted:** Not Highlight

253 ~~contributing only 5 % of about 80 % of petroleum consumption (Acheampong & Menyeh, 2022;~~
254 ~~Anyars & Adabor, 2023).~~ This exposes Ghana’s economy to shocks from global oil price with
255 adverse repercussions economic development
257 The 2015 Sustainable Development Goals (SDGs) (1, 2, & 14) seek to eradicate poverty,
258 hunger, and all forms of food insecurity and malnutrition by 2030, including the sustainable
259 management of marine and coastal ecosystems. However, the challenges to ending poverty,
260 hunger, food insecurity, and malnutrition keep growing (FAO, 2022). The Russia-Ukraine conflict,
261 the high cost of living, the COVID-19 pandemic, and climate change are affecting the livelihoods
262 of people across the world (Parker & Tyedmers, 2015; Bennett et al., 2020). The COVID-19
263 pandemic exposed the fragilities in agrifood systems and the inequities driving further increases
264 in world hunger and severe food insecurity (FAO, 2022). Prior to the recent global oil price surge,
265 the covid 19 pandemic disrupted the global fisheries supply value chains, leading to higher aquatic
266 food prices of which Ghana was no exception (Owusu et al., 2022; Bennett et al., 2020; Marcusi
267 et al., 2022). About 820 million people worldwide suffer from malnutrition, and another 736
268 million live in extreme poverty, most of which are in developing countries (Dalheimer et al., 2021).

**Formatted:** Indent: First line: 0.3"

269 Ghana is experiencing a significant increasing inequality, uncertainties in fish availability, and
270 unsustainable fisheries management (Hasselberg et al., 2020). Demand for fish exceeds the
271 domestic supply, and the shortfall is covered by approximately 200,000 tons of imports (Ministry
272 of Fisheries and Aquaculture Development, 2016). The total export for 2021 was 67,786.03mt
273 (provisional) in 2021 and 37,156.70mt in 2020, a significant 30,629.33mt increase. The country
274 imported 273,382.32mt in 2021 as against 193,226.87mt in 2020, which saw an increase of
275 80,155.45mt (Fisheries Commission, 2022).
276 Ghana has a coastline of 550km along the Gulf of Guinea. The fisheries sector consists of
277 marine capture fisheries, inland fisheries, and aquaculture. The marine fisheries sector comprises
278 four subsectors: artisanal or small-scale, semi-industrial or inshore, industrial, and tuna fisheries (
279 MoFAD 2016). Marine fishing is an important traditional economic activity of the coastal
280 communities, contributing over 80 percent of the total fish catch ( Kwadzo, 2022; Ofori-Danson
281 et al.2019 ). Marine fisheries resources include small pelagic, large pelagic (Tunas), and demersal
282 species. The most important small pelagic include round sardinellas, flat sardinellas, anchovy,
283 chub, and horse mackerels, contributing 60-70% of total marine fish production (Fisheries
284 Commission, 2022). The small pelagic contributes immensely to food and nutrition security. The
285 most important demersal species include sea breams, red snappers, groupers, grunts, croakers,
286 cephalopods, and shrimps and, together with the large pelagic (Bigeye, Yellowfin, and Skipjack),
287 contribute significantly to foreign exchange earnings (Fisheries Commission, 2022).
288 The t~~T~~otal d~~D~~omestic p~~P~~roduction by artisanal canoe in 2021 amounted to 223,088.61
289 ~~628,617.52~~mt compared to 181498.81~~442,903~~mt in 2020 (Figure 3). Overall,, representing an
290 ~~average percentage increase of 42~~. the m~~M~~arine sector recorded 393,970.01mt in 2021 against
291 297,976mt in 2020, representing a 32% increase (Fisheries Commission, 2022).
292 Fig~~IG~~ 3. Total marine fish production by artisanal canoe 2000-2021 (metric tons)
293
294 Two coastal communities located in the Central Region were selected for this study. ~~(Figure~~
295 ~~4). Despite their significant contribution to the local fisheries industry, there has not been any~~
296 ~~study to examine the increase in fuel prices and its repercussions on fisheries’ livelihoods~~.

**Formatted:** Indent: First line: 0.3"

**Formatted:** Font: 16 pt

**Formatted:** Justified, Indent: First line: 0.3", Line spacing: 1.5 lines

**Formatted:** Indent: First line: 0.3"

**Formatted:** Left, Indent: First line: 0.3"

297 Winneba is a coastal town in southern Ghana. It is located in the Central Region and serves
298 as the capital of the Effutu Municipal ~~(Figure 4)~~. Winneba lies on latitude 5°22'01.5"N and
299 longitude 0°37'52.2"W along the Gulf of Guinea. Winneba is situated 56 km west of Accra,
300 Ghana’s capital city, and 140 km from Cape Coast (Ankrah, 2018). Winneba is a vibrant fishing
301 area with five landing sites, 4270 fishermen, and 436 canoes (MoFAD, 2016). The main fishing
302 grounds include Eyipey, Winneba, Esuakyir, Warabeba, and Akosua Village. Mackerel, kingfish,
303 tuna, sea bream, and herring are some fish species caught here ( MoFAD, 2016 ). Besides marine
304 fishing, the Muni Lagoon is an important fishing ground for most fisherfolk (Okyere et al.,
305 2023). Three finfish and eight shellfish species are found in the lagoon. The dominant species is
306 black chin tilapia, *Sarotherodon Melanotheron* (Cichlidae) (Okyere et al., 2023). The men are
307 responsible for fishing, while the women are involved in fish processing, trading, and marketing.
309 Apam is a coastal town located along the Atlantic Ocean in Ghana. It is the capital of the
310 Gomoa West District in the Central Region of Ghana. It is approximately 68 km east of the Central
311 Regional capital, Cape Coast, and about 69 km southwest of Accra (Agyemang & Amissah, 2019).

312	It lies on the coordinates 5°18'17.91"N, 0°42'34.89"W, and 5°14'47.33"N, 0°46'35.27"W.

313 According to MoFAD ( 2016), ten landing sites, 4,062 fishermen, and 298 canoes are registered
314 in the community to support the fishing industry. The main fishing season in Apam is between
315 August and December. Some of the commonly caught fish include threadfin (*Galeoides*
316 *decadactylus*) and burrito (*Brachydeuterus auritus)* (MoFAD, 2016).
318 **~~Fig 4. Map of the study area in a regional and national context~~**

320	**Research Methodology**

321 Data collection
322 The study used a mixed-method approach to investigate the effects of fuel price hikes on the
323 livelihoods of coastal fisherfolk. The empirical data consists of a questionnaire survey of 3~~2~~20
324 SSF-dependent households and 20 interviews with stakeholders in the fisheries sector. The use of

**Formatted:** Justified, Line spacing: 1.5 lines

325 both quantitative and qualitative approaches provided a better comprehension of the research
326 problem and facilitated cross-validation and triangulation. The surveys and interviews were
327 conducted between July and August 2022 in the communities of Winneba and Apam in the Central
328 Region of Ghana. In-depth semi-structured interviews (N=20) were conducted with community
329 leaders (n=15), government officials (n=4), and NGO officials (n=1) to gain insights into the
330 experiences of fisherfolk concerning fuel price hikes. The data was gathered in the language the
331 respondent best understood after obtaining informed consent. The languages used included
332 English, Fante, and Twi. The questionnaire and interview guide were designed in English;
333 however, when a respondent did not understand the questions, the local languages mentioned
334 above were used to explain the questions. To ensure interviewer reliability for consistent data
335 collection, a common standard for selecting and training the research assistants was set. All the
336 research assistants had fundamentals in conducting research and fluency in English, Fanti, and Twi
337 languages. The research assistants were trained in the data collection processes and had sufficient
338 knowledge of the issues under investigation.
339 The household questionnaire covered three broad thematic areas: socioeconomic and
340 demographic information, effects of fuel price hikes, and coping mechanisms. The questionnaire
341 included closed-ended, open-ended, and multiple-choice questions. The open-ended questions
342 allowed the respondents to freely talk about their experiences and knowledge relevant to the local
343 fishing industry and other relevant issues concerning the topic under investigation. The survey
344 lasted between 50 minutes to an hour on average per person. The major themes in the interview
345 guide were the socio-demographic information on respondents, fuel prices and fishing operations,
346 and the coping strategies deployed to mitigate the effects.
347 Sampling method and sample size
348 Quantitative and qualitative sample sizes were determined using probability and non-probability
349 sampling procedures. Simple random and purposive sampling techniques were employed in
350 selecting respondents for the household survey and interviews. According to the 2016 national
351 canoe frame survey, there were 4270 fishermen in Winneba and 4062 in Apam. The number of
352 fishermen in the study communities was similar during the survey. Information on the population
353 of fishermen was sourced from the Chief fisherman to constitute the sample frame. A total of 3~~2~~20
354 fisherfolk were engaged in the household survey~~research~~, including 170~~20~~ randomly selected
355 fishers engaged in small-scale commercial fishing from Winneba and 15~~0~~0 from Apam. Random
356 sampling enables each individual in the population to have an equal chance of being selected
357 (Creswell, 2014). Based on information provided by the chief fishermen that showed the presence

**Formatted:** Font: (Default) Times New Roman, 12 pt

358 4270 fishermen in Winneba and 4062 in Apam. The sample size was therefore determined using
359 Yamane formula:

**Formatted:** Font: English (United Kingdom)

Sn =	𝑁 1+𝑁(𝑒)^2^

361 Where:
362 Sn = sample size
363 N = population size (8,332)
364 e = margin of error

Sn =	8332

1+8332(0.05)^2^

| 366 | = | 8332 | | | | |
| --- | --- | --- | --- | --- | --- | --- |
|  |  | 21.83 | | | | |
| 367 | = 381.67 | | | | |  |
| 368 | =382 approximately | | | | |  |
| 369 | **Table 1. Sample structure for selecting fisherfolk** | | | | | **Formatted:** Font: Bold |
|  |  | | | | |  |
|  | **Study areas** | | **Population** | **Proportion** | **Sample size** | |
|  | Winneba | | 4,270 | 51% | 195 | |
|  | Apam | | 4,062 | 49% | 187 | |
| 370 | **Total** | | **8332** | **100** | **382** | |

371 The sample sizes were proportional to the population of each area, with Winneba being slightly

**Formatted:** Indent: First line: 0"

372 larger (Table 1). However, 320 (84 percent) of the 382 respondents provided usable information
373 for the study. The remaining 14% either did not complete the survey or declined to do so. Even
374 though not up to the required sample size, the data is still large enough to ensure statistical validity
375 for comparison between the two areas. The sizes are within a reasonable range to detect significant
376 differences or trends in the population without introducing major biases.

**Formatted:** Font: (Default) Times New Roman, 12 pt

377 The purposive sampling technique was used to select community leaders, government

**Formatted:** Indent: First line: 0.3"

378 officials, and NGO representatives. In-depth semi-structured interviews (N=20) were conducted
379 with community leaders (n=15), government officials (n=4), and NGO officials (n=1) to gain
380 insights into the experiences of fisherfolk concerning fuel price hikes. These stakeholders were
381 considered to have the requisite knowledge and experience with fishing and other related activities
382 in the studied communities. The goal here is to acquire an in-depth understanding of the subject of
383 the study from participants viewpoint. The sample size was determined upon saturation point (see
384 Hay 2010). These sampling techniques are efficient data-gathering methods and have been used
385 by several authors to investigate fisheries livelihoods and coastal development in West Africa
386 (Penny et al., 2017; Adjei & Overå, 2019; Marquette et al., 2002).
387 Data analysis
388 SPSS Version 23.0 and Excel were used to analyze the results from the household surveys.

**Formatted:** Indent: First line: 0.3"

389 The socio-demographic characteristics of the respondents were presented in frequency tables. The
390 effects of the fuel price hike, and coping strategies were presented in tables and charts. The
391 interviews were audio-recorded with the respondents' permission and later translated verbatim.
392 The transcribed data was manually coded and organized into relevant themes based on the study's
393 objectives and components of SLA used in the study. Qualitative content analysis technique was
394 used to interpret meaning from the content of text data (Nowell et al., 2017). The author read
395 through the transcripts severally to fully comprehend responses given by research participants and
396 highlighted particular words from the text that appear to capture critical thoughts and answer the
397 research questions. Selected narratives from the in-depth interviews were then presented as direct
398 quotations to illustrate key findings. Direct field observation of the coastal environments and
399 fishing activities further provided a depth of information. Participant observation was also
400 conducted at the fish markets and landing beaches. Information related to the organization of
401 fisheries and other related activities, such as trading and processing, were recorded. These
402 observations provided insights into the social and economic environments where fishers perform
403 daily economic activities and related social functions. Secondary data was collected from
404 published and non-published reports, online newspaper articles, and internet sources to support the
405 analysis of the study.

406	**3~~4~~.Results**

**Formatted:** Font: 18 pt

407 Table 2~~1~~ presents an overview of selected socio-demographic features of the two study

**Formatted:** Indent: First line: 0.3"

408 communities. The study communities in Apam recorded the highest household number, with
409 33.3% of respondents indicating they have more than 10 members in their household. Apam has
410 the highest number of fishers with no formal education (43.2%). Across the study areas, the high
411 cost of living was the topmost perceived problem ( Table 2~~1~~). Research participants indicated that
412 the increase in fuel prices was the major cause of the high cost of living. The majority of the
413 fisherfolk in the study areas have no alternative work apart from fishing ( Table 3~~2~~).
419 **Table 2~~1~~. Characteristics of the study sites and persons interviewed (n)=3~~2~~20**

| Variable | Value | APAM | WINNEBA |
| --- | --- | --- | --- |
| AGE | BELOW 20 | 3.8 | 5.9 |
|  | 20-39 YEARS | 44.2 | 43.1 |
|  | 40-59 YEARS | 38.5 | 37.3 |
|  | ABOVE 60 | 13.5 | 13.7 |
| MARITAL STATUS |  |  |  |
|  | SINGLE | 17.3 | 29.4 |
|  | MARRIED | 59.6 | 45.1 |
|  | DIVORCED | 13.5 | 13.7 |
|  | SEPARATED | 9.6 | 11.8 |

420

| EDUCATIONAL LEVEL |  | | |
| --- | --- | --- | --- |
|  | NO FORMAL | 43.2 | 28.8 |
|  | EDUCATION  ELEMENTARY | 37.2 | 50.0 |
|  | SECONDARY | 17.6 | 21.2 |
|  | TERTIARY | 0.0 | 0.0 |
|  | TECHNICAL | 2.0 | 0.0 |
|  | TRAINING |  |  |
| TYPE OF FISHER |  |  |  |
|  | CAPTAIN | 25.0 | 21.6 |
|  | CREW MEMBER | 40.4 | 41.1 |
|  | BOAT/CANOE | 34.6 | 37.3 |
|  | OWNER |  |  |
| NUMBER OF PEOPLE IN YOUR | LESS THAN 5 | 13.4 | 15.7 |
| HOUSEHOLD | 6 - 10 | 61.5 | 51.0 |
|  | MORE THAN 10 | 25~~33~~.13 | 25.1 |
| WHAT IS YOUR AVERAGE MONTHLY | 0 - 199 | 0.0 | 0.0 |
| INCOME FROM FISHING? | 200 - 399 | 5.7 | 3.9 |
|  | 400 - 599 | 26.9 | 27.5 |
|  | 600 - 799 | 23.2 | 23.5 |
|  | MORE THAN 800 | 44.2 | 45.1 |
| WHAT IS YOUR AVERAGE MONTHLY | 0 - 199 | 76.9 | 78.4 |
| INCOME FROM OTHER INCOME- | 200 - 399 | 17.4 | 15.7 |
| GENERATING ACTIVITIES? | 400 - 599 | 5.7 | 2.0 |
|  | 600- 799 | 0.0 | 0.0 |
|  | MORE THAN 800 | 0.0 | 2.0 |
| TOP TWO PROBLEMS IN THE | High cost of living | 100.0 | 100.0 |

423

COMMUNITY AT THE MOMENT

Unemployment

50.0

70.0

428 Exchange rate: USD 1 = GHS 9 as of August 2022
432
449 **Table 3~~2~~. Key survey results**

**Formatted:** Font: 16 pt

|  | Variable | Value | APAM | WINNEBA |
| --- | --- | --- | --- | --- |
|  | Purpose of catch | Commercial Domestic | 100  - | 100  - |
|  |  | Increase | 3.0 | 2.0 |
|  | Catch quantity changes (over the past 5 years) | No change  Decrease | 5.0  92.0 | 4.0  94.0 |
|  |  | Higher | 2.0 | 3.0 |
|  | Income changes (over the past 5 years) | No change | 6.0 | 4.0 |
|  |  | Lower | 92.0 | 93.0 |
|  | How long have you been fishing? | Less than 5 years | 3.8 | 5.9 |
|  |  | 5 to 10 years | 13.5 | 15.7 |
|  |  | 11 to 15 years | 36.5 | 31.4 |
|  |  | Over 15 years | 46.2 | 47.0 |
|  | Do you have other jobs besides fishing? | Yes | 5.7 | 21.6 |
|  |  | No | 94.2 | 78.5 |
|  | Has the increase in fuel prices in any way affected your business operation? | Yes | 100 | 98.1 |
|  |  | No | 0.0 | 1.9 |
| 451 |  |  |  |  |
| 452 |  |  |  |  |
| 453 **4.1 Perceived effects of high fuel price on fisheries livelihoods** | | | | |

454	Four main issues were identified concerning how increased fuel prices affected the livelihoods

455	and well-being of coastal fisherfolk. These include an increase in expenses, reduced frequency of

456	fishing, an upsurge in social vices, and declining small-scale fisheries opportunities (Figure 4)..

457

458	Figure 4. Perceived effects of high fuel price on fisherfolk livelihoods.

**Formatted:** Font: Bold

459

460

461	Table 4~~3~~. Impacts of the high fuel cost on livelihood assets of fisherfolk

462

**Capital asset	Perceived influence of high cost of fuel on livelihood ~~assests~~assets**

Natural capital	Limited fishing operation and low fish stock undermines traditional livelihoods.

Human capital	Reduced fish consumption undermines nutritional and food security; frustration, anger, and anxiety affect the health and well-being of fishing households. No provision for training or capacity building for fisherfolk to undertake alternate livelihoods

Financial capital	High cost of fuel, increased cost of living, no additional sources of finances to support daily subsistence, and low savings affect investment into other economic sectors.

Physical capital	Loss of material properties due to theft, limited ownership of productive assets such as canoes and nets.

Social capital	Weakening social capital, increases social vices erodes communities' collective action, and increases social tension.

463

464 The results from the household survey, coupled with the interviews, revealed that there has been
465 a substantial increase in expenses concerning fishing operations. The Government of Ghana
466 distributes premix fuel to coastal fishing communities at a much-subsidized price. Premix fuel
467 subsidy was introduced in Ghana's small-scale fisheries in the 1990s to deal with the high cost of
468 fuel and reduce the socioeconomic burden on the poor and vulnerable fisherfolk in coastal
469 communities ( Okyere et al., 2020; Owusu & Adjei, 2021).The subsidized premix fuel, which
470 was sold at GHS10 per gallon, has been increased to GHS25 per gallon because of the rising oil
471 prices (Addo, 2023). The majority of fisherfolk reported that the increase in fuel prices has
472 negatively affected the supply of premix fuel. Fishers have to wait for several weeks and months
473 without premix fuel supply, and the only option is to buy expensive petrol for fishing activities:
474 *Since the price of fuel started increasing, the supply of premix fuel has not been frequent. The price*
475 *of the premix fuel has also increased, which has rendered most of us unable to undertake our*
476 *fishing activities, especially those who embark on fishing activities at night.*
477 (Canoe owner, Apam.)
478 *The rise in the price of petrol has brought a lot of negative effects on the fishermen. Those who*

**Formatted:** Font: Italic

479 *sell the petrol to us don't even get some to buy for us to purchase from them. This has caused an*
480 *increase in the price of the premix fuel, because if you want to buy the fuel unless you resort to*

**Formatted:** Font: Italic

481 *third parties rather than the normal channel of buying it.*
482 ( Chief Fisher, Apam)
483 Fisherfolk emphasized that even though the supply of the premix fuel was irregular, the recent fuel
484 price hikes have worsened the situation. The majority of the fisherfolk reported that the increase
485 in fuel prices has reduced the frequency of fishing operations, resulting in low catch and income.
486 Interviewed canoe owners reported spending more on fuel and food for their crew members.
487 The following extracts from the interviews with fishers aptly speak to the findings presented
488 above:
489 *I have lost about GHS2000 this month. Anytime there is an increase in fuel prices, fishermen suffer*
490 *a lot. The high fuel prices have affected everything. A small gallon of petrol is sold at GHS40 while*
491 *the bigger gallon is over GHS250. Two of my canoes have been packed because of the high fuel*
492 *price. I do not have any additional work besides fishing.*
493 (Canoe owner and Fisher, Winneba)
494 *The increases in fuel price have impacted us negatively in many ways. Most of us now incur losses*
495 *as a result of the increment. We used to fish five times during the week; however, because of the*
496 *high fuel cost, it has been reduced to only two fishing trips. The quantity of fuel determines the*
497 *duration of fishing. Our canoe owner says he cannot afford more fuel because of the higher prices.*
498 (Fisher, Apam)
499 Responses generated from the interviews with local fisherfolk—fishers, canoe owners, and local
500 leaders revealed that increases in fuel prices have a negative implication on fishing households’
501 financial capital development. . Fuel constitutes a substantial component of the cost of fishing;
502 globally, about 30-50% of fishing expenditure is associated with fuel (Sumaila, 2008). In
503 developing countries, fuel constitutes about 50% of fishing expenditure within small-scale
504 fisheries (Parker & Tyedmers, 2015; Sumaila, 2008).
505 The increases in fuel prices affect other fishery-related businesses, such as processing and trading.
506 Interview with some of the fishmongers and traders indicates that the increase in fuel prices has
507 affected the quantity of fish supply and the price of fish. This point was echoed by the ~~a~~ fish
508 processors:
509 *The increase in fuel prices has led to the high cost of fish, which has brought us economic hardship.*
510 *We are unable to buy large quantities of fish as we did previously. This has affected our income,*
511 *and we struggle to care for our families.*
512 (Fish processor and trader, Winneba)
513 *The inflow of petrol to the coast is not frequent and this has compelled many fishermen to buy fuel*

**Formatted:** Font: Italic

514 *from ‘middlemen’ at expensive cost. The prices of fuel has caused an increase in the prices of fish.*

**Formatted:** Font: Italic

**Formatted:** Font: Italic

515 *About 20 years ago, when the fisheries business was good, l was able to establish a small trading*
516 *shop. However, in recent times, we barely make any profit from the fishery business to support our*

**Formatted:** Font: Italic

517 *families and invest in other businesses. I'm appealing to the government to reduce the price of*

**Formatted:** Font: Italic

518 *fuel and increase the supply for the fishermen to facilitate their work.*

**Formatted:** Font: Italic

519 ( Fish processor and trader, Apam)

**Formatted:** Font: Not Italic

520 The ~~Surveyed~~ fishers reported that they sell fish at a much higher price because of increases in the
521 prices of fuel:
522 *We sell fish to the women, and they complain it is expensive, but we have to pay for the high fuel*
523 *cost. The rise in the price of petrol has affected us in so many ways, and those who even sell petrol*
524 *to us do not even get some to buy for us to purchase, which has worsened the price of the premix*
525 *fuel. We used to buy a drum/gallon of premix fuel at GHS700, but now it is sold at GHS1200-1300*.
526 (Fisher, Apam)
527 Results from the household surveys and interviews show that fisherfolk are currently undergoing
528 severe economic hardship. The increased expenditure on fishing inputs such as fuel remains a
529 major financial burden on fishing households. A recent study by Koranteng (2022) found that a
530 box of fish sold at GHS 250 is now sold between GHS450 and GHS500. The increase in fuel prices
531 has significantly led to increases in inflation, with food prices being the most affected (GSS, 2022).
532 According to the Ghana Statistical Services (2022), prices of food items such as cereal (rice, corn,
533 millet, grains, wheat, sorghum), meat, fish and other seafood, milk, dairy products, eggs, and
534 cooking oil increased significantly.
535 Many fishing households reported that their children are not able to go to school because of a lack
536 of money to cater for their needs:
537 *I find it difficult to cater to my children’s education. Though my husband is working, he does not*
538 *earn much to take care of our children, not to mention housekeeping money from him for our*
539 *feeding. I mostly pay for my children’s school fees. However, because of financial problems, they*
540 *must stay home for some time before returning to school. Some of us barely eat once in a day.*
541 (Fish processor and trader, Apam).
542 Another participant remarked:
543 *Our children are suffering because there has been a frequent rise in the price of fuel. We are*
544 *suffering; the hardship is too much for us. They told us we would not pay school fees, but we were*
545 *paying more than the school fees. Some of my children have dropped out of school because of my*
546 *inability to pay their school fees. Many of us now rely on our mothers and buyers for survival. I*
547 *have lost much money as a result of the high cost of fuel, and this has compelled most of us to*
548 *resort to borrowing.*
549 Another social problem emerging from the fuel price hikes is the upsurge of social vices and
550 increased community tension. Many fishing households reported that ~~their money~~their money,
551 foodstuffs, and other personal properties were stolen. Many respondents revealed that the upsurge
552 in social vices has led to considerable frustration, stress, and anxiety in the community:
553 *Apart from financial problems, I go through psychological stress because I get traumatized and*
554 *think a lot about how I will survive since I do not have any other work besides fish processing.*
555 *Many have lost their jobs and livelihoods, and there has been an increase in social vices in the*
556 *community.*
557 (Fisher processor and trader, Apam)
558 Another fisher opined :
559 *The economic hardship has made some youth in the community resort to illegal ways of getting*
560 *money to cater for themselves and their family. This has led to robbery in our neighborhoods.*
561 *Since this town has no jobs, we are appealing to the government to create jobs for us.*
562 The empirical findings from this study suggest that the fuel price hikes resulted in the loss of
563 income and heightened social tension due to increased social vices. This negatively impacts the
564 human and physical capital development in coastal communities. Increased anxiety and financial
565 stress affect the well-being and health of fisherfolk in coastal communities.
566 **5.2 Livelihood Coping ~~Adaptation~~ Strategies**

**Formatted:** Font: 14 pt

**Formatted:** Font: 14 pt

567 The livelihood strategies reported by fisherfolk include dependency on savings, borrowing, petty
568 trading, and farming. 60 % of surveyed fisherfolk in Apam depended on their savings and
569 borrowing to care for their families, while 50% of fisherfolk in Winneba depended on their savings
570 and borrowing (Figure 5)..
571

**Formatted:** Justified, Line spacing: 1.5 lines

572 **Fig~~ure~~ 5~~5~~. Coping strategies (based on fishers’ multiple-choice responses, % of fishers;’ n=3~~2~~20).**
573 The fishers borrow money from canoe owners, friends and families, and fish traders. They also
574 sometimes buy foodstuffs on credit from fish traders, processors, and other market women. Most
575 fishers reported that they defray their debt with fish during the major~~bumper~~ fishing~~harves~~t season:
576 *I borrow money from the fishmongers, and l always pay back with fish. Sometimes, we buy food*
577 *and other commodities on credit, hoping to repay them during the bumper harvest season.*
578 *However, a major challenge is that because we are not getting a lot of fish during major fishing*
579 *seasons, it becomes difficult to pay back our debt*. *This has resulted in many people refusing to*
580 *lend us money and food items..*
581 (Fisher, Winneba)
582 Some fisherfolk also engaged in petty trading, such as selling coconut and used clothing. Others
583 engaged in handicrafts such as mat weaving;
584 *I save from the little I get when I sell the fish and engage in other trading activities that involve*
585 *selling used clothing to help me care for my children because I am a single parent.*
586 (Fish processor and trader, Apam)
587 In interviews, some fish traders mentioned that the lack of capital to expand the business remains
588 a major challenge to sustain their operation. Moreover, the demand for items is not encouraging,
589 making it difficult to make profit.
590 Farming is practiced by just 10 % of fisherfolk in Winneba. Some of the common crops cultivated
591 are maize and cassava. In Apam, no fisherfolk reported any farming activity. Another major
592 important coping strategy reported by fisherfolk is migration. Fishers in both study communities
593 reported that they regularly travel to fish in other coastal communities:
594 ~~The Muni lagoon in Winneba served as a major fishing ground for fisherfolk during the closed~~
595 ~~fishing season. The closed fishing season has been enforced for a period of one month every year~~
596 ~~since 2019 for the SSF. The no-fishing zone covers the entire 228,000km² Exclusive Economic~~
597 ~~Zone (EEZ) of Ghana’s coast ( Kwadzo, 2022). 40% of surveyed fisherfolk in Winneba reported~~
598 ~~fishing in the Muni lagoon and other nearby coastal waters~~:
599 *I travelled to Weija to fish, we sometimes do not get a good catch, and the fuel cost affects us, but* 600 *we do not have any job apart from fishing. We also sometimes travel to fish at Elmina and Cape* 601 *Coast to increase our catch.*

602	( Fisher, Winneba)

603	There are no jobs in this area, the only job we have is fishing. We regularly made enquiries from 604	other coastal areas to ascertain where there are abundant catch. When we hear of abundant catch 605 in other areas , l mobilize my crew and travel to those areas to catch fish.

606	(Canoe owner , Apam)

607	Migrating to fish is an important coping/adaptation strategy among fisherfolk in Ghana

608	(Marquette et al., 2002; Owusu, 2019).

609	Other coping strategies reported include reducing the frequency and quantity of food consumption. 610	Fisherfolk reported that the reduction in the frequency and quantity of food consumed preceded 611	the fuel price hikes. However, it became more pronounced since fuel prices started increasing. 612	50% of respondents in Apam indicated that they have reduced the frequency of food consumption 613	whilst 40 % of respondents in Winneba indicated that they have also reduced the frequency of food 614 consumption.

615	5. **Discussion**

**Formatted:** Font: 18 pt

616	This paper sought to show how recent fuel price increases affect small-scale fisheries’

617	livelihoods and marine sustainability. The study also explores the various coping strategies

618	deployed by fisherfolk in response to rising fuel prices. Increases in expenses, decreased fishing 619	frequency, upsurges in social vices, and declining small-scale fisheries opportunities were found 620	to be the main effects of high fuel prices on fisheries livelihoods. ~~The findings from the study~~ 621	~~suggest global economic shocks such as oil price hikes have local economic implications that~~ 622	~~negatively affect the well-being and health of coastal fishing communities~~. Fuel price hikes have 623	negatively impacted fishing households' financial, human, and social capital assets.

624	The financial capital assets of coastal fishing communities have deteriorated because of the 625	fuel price increases. The high fuel cost has led to decreased fishing frequency resulting~~ed~~ in low 626	fish stock, and income (Table 4).. Households that entirely depend on fishing and its related 627	activities are most affected as they do not have any viable alternative sources of income (Table 628	3~~2~~). Households with fewer livelihood options are most vulnerable to external shocks and are 629	susceptible to falling back into poverty (Andriesse, 2018; Owusu & Andriesse, 2022). The 630	increases in fuel prices have also resulted in high cost of fish at the various landing sites. This has 631	brought financial burden on women, as they are unable to buy large quantities of fish for processing 632	and trading. Previous studies show that women are among the most vulnerable in coastal fishing 633	communities due to their limited ownership of fishing assets and participation in fishing work ( 634 Danso-Wiredu, 2018; Adusah-Karikari, 2015). Fishers consider fishing to be an important

**Formatted:** Indent: First line: 0.3"

**Formatted:** Indent: First line: 0.3"

635	occupation that provides their primary source of income and considering that there are no viable 636	alternative income-generating activities, the high fuel prices impose harsh economic conditions 637	and undermine traditional livelihoods. The small-scale fisheries in West Africa have suffered from 638	rapidly declining marine fish landings, resulting in decreased income and severe economic 639 hardships (Yang et al., 2019; World Bank, 2016; Adusah-Karikari, 2015). The decrease in income 640	and declining small-scale fisheries opportunities has led to disruption in the education of fisherfolk 641	children. Fisherfolk are unable to pay their children school fees leading to school dropouts or 642 temporarily stopping school.

643	The global oil price shock has exposed fundamental structural weaknesses in Ghana’s economy 644	(Acheampong & Menyeh, 2022). Despite being a net exporter of crude oil, the country has failed 645	to cushion the citizenry against escalating global oil prices. This has adversely affected the 646	livelihoods and economic condition of coastal fishing communities. As Anyars & Adabor (2023) 647	noted, increasing oil prices generates revenue for oil-exporting countries; however, it also poses a 648	potential threat of causing inflation, recession, low growth, high food prices, and low development 649	for oil-importing countries. Ghana imports about 95 % of oil, with local production contributing 650	only 5 % of about 80 % of petroleum consumption (Acheampong & Menyeh, 2022; Anyars & 651 Adabor, 2023). This exposes Ghana’s economy to shocks from global oil price with adverse 652 repercussions economic development

653	The findings from this study also show that there has been an	upsurge in social vices. The 654	upsurge in social vices has led to~~The~~ high levels of frustration, anxiety, and stress among the 655	fisherfolk.~~worry experienced by fishing households are manifestations of the shock and stress~~ 656	~~induced by fuel price hikes (Figure 2)~~. Fisherfolk are in fear and panic because of the persistent 657	loss of personal properties.~~fuel price hikes. They are worried about getting their daily needs and~~ 658 ~~caring for their families and children’s education. They were frustrated about the persistent loss of~~ 659	~~items in the community and are angry as they could not immediately provide money for their daily~~ 660	~~expenses~~. As ~~This new development comes when~~ fishing communities are recovering from the 661	fear, panic, and anxiety induced by the COVID-19 pandemic on their livelihoods ( Bennett et al., 662	2021; Marcusi et al. 2022), the recent fuel price hikes has worsened living conditions of fisherfolk. 663 The findings from this study reinforces the need to pay more attention to the psychological and

**Formatted:** Indent: First line: 0.3"

664	mental health needs of coastal fisherfolk. ~~(Bennett et al., 2021; Marcusi et al., 2022).~~As noted by 665	Owusu & Andriese (2022)	~~Traditional~~ village-based livelihood analysis has overly focused on 666	developing financial and social capital with little emphasis on the well-being and health component 667	of rural folks. ~~human capital (Owusu & Andriesse, 2022).~~ Without good health, fisherfolk cannot 668 perform their daily economic activities. Increased financial stress and anxiety emanating from fuel 669 price shocks could trigger other diseases that further impoverish the health status of fisherfolk. 670	~~Livelihood assets should be considered as interdependent. The findings from this study sugges~~t 671 ~~the need to pay more attention to the well-being and health and the overall human capital~~ 672 ~~development in coastal fishing communities~~.

673	Despite the uncertainties that fisherfolk have to face, the increases in fuel prices can indirectly 674 contribute towards the restoration of depleted marine fish stock through a reduction in fishing 675	pressure and overcapacity. Several scholars have	concluded~~alluded~~ that Ghana's fisheries 676	resources are overfished and overexploited (Atta-Mills, 2014; Finegold et al., 2010; Hasseberg et 677	al., 2020). The rise in fuel prices, coupled with the increases in the prices of subsidized premix 678 fuel, can translate into positive marine ecological gains in the long term despite the current 679	socioeconomic hardship experienced by coastal fishing communities ( Sumaila et al. 2008). 680	~~However, this largely depends on the level of fuel subsidies provided by the government, as~~ 681 ~~previous rises in fuel prices have led to an increase in fisheries fuel subsidies in some countries~~ 682	~~(Sumaila et al., 2008). The lack of effective control of fuel subsidy distribution and fishing efforts~~ 683 ~~could be counterproductive as the profit generated from subsidies may be reinvested into the~~ 684 fishery business (Munro & Sumaila, 2002; Sumaila, 2008). Several scholars have alluded that 685	Ghana's fisheries resources are overfished and overexploited (Atta-Mills, 2014; Finegold et al., 686	2010; Hasseberg et al., 2020). The rise in fuel prices, coupled with the increases in the prices of 687 subsidized premix fuel, can translate into positive marine ecological gains in the long term despite 688 the current socioeconomic hardship experienced by coastal fishing communities.

689	The empirical findings from the study revealed that fisherfolk engage in both marine and non 690	-marine coping strategies to mitigate against the socioeconomic hardship induced by fuel price 691 hikes. The marine -based coping strategies include traveling to fish in other coastal communities.

692 The non- marine based coping strategies included farming and petty trading. However, t~~T~~he 693	various livelihood strategies deployed by fisherfolk were not enough to ameliorate their economic 694	hardship. Livelihood strategies are constantly being revised to cope with contemporary trends of 695	vulnerabilities (Natarajan et al., 2022). Individuals and households explore new forms of 696	livelihoods by expanding their non-fisheries-related income sources while maintaining their base 697	in fisheries (Bryceson, 2002). The findings show that fisherfolk’s alternative sources of income 698 could hardly sustain them as majority depends solely on the fisheries (Table 3).

699	~~Understanding how people succeed or fail in sustaining their livelihoods in the face of oil~~ 700	~~price shocks can help design policies and interventions to assist peoples' coping and adaptive~~ 701 strategies (Allison & Horemans, 2006; Anyars & Adabor, 2023). The various livelihood strategies 702 deployed by fisherfolk were not enough to ameliorate their economic hardship. The findings show 703 that fisherfolk’s alternative sources of income could hardly sustain them. Livelihood strategies are 704	~~constantly being revised to cope with contemporary trends of vulnerabilities (Natarajan et al.~~, 705 ~~2022). Individuals and households explore new forms of livelihoods by expanding their non-~~ 706 ~~fisheries-related income sources while maintaining their base in fisheries (Bryceson, 2002)~~.

707	Understanding how people succeed or fail in sustaining their livelihoods in the face of global 708 oil price shocks can help design policies and interventions to assist peoples' coping strategies 709	(Allison & Horemans, 2006; Anyars & Adabor, 2023). This study provides two implications for 710 SSF-related policies in coastal Ghana: The introduction of social intervention programs and 711	supplementary livelihoods programs. There should be better-coordinated management strategies 712	by the national government and concerned stakeholders to provide incentives that contribute 713	directly to improving coastal fisherfolk livelihood conditions. In this regard, some fund ~~percentage~~ 714	~~of oil revenue~~ can be set aside and deposited into local welfare funds to compensate vulnerable 715	fishing households during global oil price shocks. . It is anticipated that this type of compensation 716	package and incentives can allay coastal communities’ fears and worries about persistent fuel price 717	increases. Cash and non-cash payments are increasingly used in marine resource management to 718	protect fishery stocks and compensate for income loss (Bladon et al., 2018; Correa et al., 2014). 719 For instance, in Bangladesh and the Brazilian Amazon, fishers receive food relief items, direct 720 cash transfers, and support for alternative income-generating activities (Correa et al., 2014; Islam

721	et al., 2016). Involved government agencies should demonstrate more political will to improve 722	human and financial capital development in coastal fishing communities by -improving access to 723	education, credit, and healthcare facilities. Specific programs that target training, improved access 724	to credit, and education on savings mechanisms would improve coastal communities' human and 725	financial status. The findings show some moderate number of fisherfolk are already engaged in 726	various petty trading activities	The government can therefore introduce interest-free loans to 727	support fisherfolk who are already engaged in small businesses. Providing business training 728	opportunities could also improve the management of finances. The provision of supplementary 729	livelihoods could improve fisherfolk’s income and well-being. The government must create the 730	enabling conditions -by providing the necessary facilities and logistics to train and enhance the 731	capacity	of	fisherfolk. The	study	recommends	aquaculture	training	for	coastal	fishing 732	communities. The training on best practices in small-scale aquaculture will be relevant to women 733	in the coastal communities, especially those directly connected to the fishing industry, as women 734 are not allowed to go to sea in traditional Ghanaian fisheries ( Danso-Wiredu, 2018).

**Formatted:** Font: (Default) Times New Roman, 12 pt

**Formatted:** Font: (Default) Times New Roman, 12 pt

735

**Formatted:** Font:

736	~~Fishing in lagoons and other inland water bodies enabled fishers to sustain their livelihoods~~. 737 ~~However, fishing in lagoons and other inland water bodies as a coping strategy during closed~~ 738	~~fishing season can undermine the ecological outcomes of conservation as fishers may intensify~~ 739	~~fishing efforts by changing gears to target different species or adopt illegal fishing methods to~~ 740	~~catch more fish (Perry & Sumaila, 2007). Overexploitation of coastal lagoon fisheries is~~ 741 ~~counterproductive for rebuilding depleted fish stock.~~

**Formatted:** Indent: First line: 0.3"

742	**6. Conclusion**

**Formatted:** Font: 18 pt

743	This paper investigated the effects of fuel price increases on the small-scale fishery’s livelihoods 744	and marine sustainability in coastal communities in Ghana. The paper applied the SLA as a 745 theoretical framework to analyze artisanal fisherfolk's vulnerability, capital assets, livelihood 746	strategies, and outcomes. Increase in expenses, reduced frequency of fishing, an upsurge in social 747	vices, and declining small-scale fisheries opportunities were found to be the main effects of fuel 748 price hikes on fisherfolk livelihoods. The results reveal that fisherfolk experienced financial,

749	economic, emotional and psychological shocks due to the high cost of fuel. Dependency on 750	savings, borrowing, petty trading, migration and farming were found to the main coping strategies. 751 However, the various livelihood coping strategies deployed by fisherfolk were not sufficient to 752 ameliorate their economic hardship.

753	~~The results reveal that fisherfolk experienced decreased catch and low income because of the fuel~~ 754	~~price hikes. The fuel price hikes were also associated with various emotional and psychologica~~l 755 ~~shocks such as fear, anxiety, and depression, which undermined the well-being and health of~~ 756	~~fishing families. Coastal communities are not homogenous concerning coping strategies~~. ~~The~~ 757 ~~presence of a lagoon in Winneba gave more fisheries-related opportunities to fisherfolk during the~~ 758 ~~closed fishing season than in other coastal communities. It is, therefore, essential to protect coastal~~ 759	~~lagoons and inland fisheries resources from overexploitation. This requires some regulatory~~ 760	~~measures to manage coastal lagoons and inland waterbodies sustainably. Fisheries managers and~~ 761	~~policymakers need to pay more attention to local area differentiation to avoid one-size-fits-for-all-~~ 762 ~~all policy prescriptions (Owusu & Andriesse, 2022).~~

763	There should be better-coordinated management strategies by the national government and 764	concerned stakeholders to provide incentives that contribute directly to improving coastal 765 fisherfolk livelihood conditions. In this regard, some percentage of oil revenue can be set aside 766	and deposited into local welfare funds to compensate vulnerable fishing households during global 767	oil price shocks. It is anticipated that this type of compensation package and incentives can allay 768	coastal communities’ fears and worries about persistent fuel price increases. Cash and non-cash 769	payments are increasingly used in marine resource management to protect fishery stocks and 770	compensate for income loss (Bladon et al., 2018; Correa et al., 2014). For instance, in Bangladesh 771	and the Brazilian Amazon, fishers receive food relief items, direct cash transfers, and support for 772 alternative income-generating activities (Correa et al., 2014; Islam et al., 2016).

773	Involved government agencies should demonstrate more political will to improve human and 774	~~financial capital development in coastal fishing communities by -improving access to education~~, 775	~~credit, and healthcare facilities. Specific programs that target training, improved access to credit~~, 776 ~~and education on savings mechanisms would improve coastal communities' human and financial~~

777	~~status. Women fisherfolk can be supported to secure small bank loans to invest in small businesses~~.

778	~~Providing business training opportunities can also improve the management of finances~~.

779	~~The provision of supplementary livelihoods could improve fisherfolk’s income and well-being.~~ 780 ~~The government must create the enabling conditions -by providing the necessary facilities and~~ 781	~~logistics to train and enhance the capacity of fisherfolk. The study recommends aquaculture~~ 782	~~training for coastal fishing communities. The training on best practices in small-scale aquaculture~~ 783	~~will be relevant to women in the coastal communities, especially those directly connected to the~~ 784	~~fishing industry, as women are not allowed to go to sea in traditional Ghanaian fisheries ( Danso-~~ 785 ~~Wiredu, 2018)~~.

786	The study agrees with Acheampong and Menyeh (2022) that the government and other concerned 787	stakeholders must expedite action on renewable energy programs to diversify the country’s energy 788 mix and improve energy security. There is a need for effective policies that can mitigate global oil 789	price shocks. The Tema oil refinery could be revamped and equipped to process local crude oil to 790 meet the high demand for petroleum products. This will help reduce economic pressures from 791 global oil price shocks in the national and local communities (Anyars & Adabor, 2023).

792	The present study focused mainly on two fishing communities in the Central Region of Ghana. 793	Thus, future studies should consider multiple case study sites of coastal areas using qualitative and 794 quantitative approaches.

795

796

797	**Acknowledgement**

798	The author wish to thank all the research assistants and research participants for making this

799	work possible.

800

801

802

803

804

805

806

807	**References**

808	Acheampong, T & Menyeh 2022. Ukraine war: how Ghana is vulnerable, and what can be done. 809	The Conversation. https://theconversation.com/ukraine-war-how-ghana-is-vulnerable- 810	and-what-can-be-done-178528

811 Addo, Z, A, B. 2023. The price of premix fuel increased from GH¢10 to GH¢25 per gallon. Citi
812 News. https://citinewsroom.com/2023/01/price-of-premix-fuel-increased-from-
813 gh%C2%A210-to-gh%C2%A225-per-gallon/
814 Adjei M, & Overå R., 2019. Opposing discourses on the offshore coexistence of the petroleum
815 industry and small-scale fisheries in Ghana. Extractive Industry & Society 6, 190–197.
816 Adom D, Sekyere PA, & Yarney L. (2019). A return to the Ghanaian cultural values of closed 817	fishing season in Ghana’s artisanal marine fishing: an essential means of restoring small 818	pelagic fish stocks. Transylvanian review of systematic and ecological research 21(3), 819	95–110.

820	Adusah-Karikari, A., 2015. Black gold in Ghana: Changing livelihoods for women in

821	communities affected by oil production. The Extractive Industries and Society, 2(1), 24-

822	32.

823 Allison, E. H., & Ellis, F., 2001. The livelihoods approach and management of small-scale
824 fisheries.Marine Policy 25(6), 377–388.
825 Allison, E. H., & Horemans, B., (2006). Putting the principles of the Sustainable Livelihoods
826 Approach into fisheries development policy and practice. Marine Policy, 30(6), 757–766.
827 Amadu I, Armah FA, Aheto DW, & Adongo CA, 2021. A study on livelihood resilience in the 828	small-scale fisheries of Ghana using a structural equation modeling approach. Ocean & 829	Coastal Management, 215, 105952.

830	Andriesse, E., (2018). Persistent fishing amidst depletion, environmental and socioeconomic

831	vulnerability in Iloilo Province, the Philippines. Ocean and Coastal Management, 157(1),

832	130–137.

833	Anyars, S. I. & Adabor, I .2023. The impact of oil price changes on inflation and disaggregated

834	inflation: Insights from Ghana. Research in Globalization, 6, 100125.

835	Atta‐Mills, J., Alder, J., & Rashid Sumaila, U., 2004. The decline of a regional fishing nation:

836	the case of Ghana and West Africa. Natural Resources Forum,

837	https://doi.org/10.1111/j.0165-0203.2004.00068.x

838 Awunyo-Vitor, D., Samanhyia, S. & Bonney, E.A. (2018). Do oil prices influence economic
839 growth in Ghana? An empirical analysis Cogent Economics & Finance, 6: 1496551.
840 Bennett, N. J., Finkbeiner,E.M, Ban,N.C., Belhabib, D., Jupiter, S.D., Kittinger, J.N.,
841 Mangubhai, S., Scholtens, J., Gill, D., & Christie , P., 2020. The COVID-19 Pandemic,
842 Small-Scale Fisheries and Coastal Fishing Communities, Coastal Management, 48:4,

843	336-347, DOI: 10.1080/08920753.2020.1766937

844 Bryceson, D.F, (2002). Multiplex livelihoods in rural Africa: Recasting the terms and conditions
845 of gainful employment. The Journal of Modern African Studies, 40(1), 1-28.
846 Carney, D., Drinkwater, M., Rusinow, T., Neefjes, K., Wanmali, S., Singh, N., 1999.
847 Livelihoods approach compared. DFID. https://[www.researchgate.net](http://www.researchgate.net/) › publication ›
848 313349342_LIVELIHOODS_A.
849 Creswell, J.W. (2014). Research Design: Qualitative, Quantitative, and Mixed Methods
850 Approaches. 4th ed. Thousand Oaks, California: SAGE Publications
851 Corrêa M, A, de Almeida., Kahn J. R., Carlos , Freitas, C, and E de Carvalho (2014). Perverse
852 incentives in fishery management: The case of the defeso in the Brazilian Amazon.
853 Ecological Economics, 106, 186-194.
854 Crawford B, Gonzales L, Amin D, Nyari-Hardi B, & Sarpong YA, 2016. Report on the Baseline 855	Survey of Small Pelagic Fishing Households along the Ghana Coast. The USAID/Ghana 856	Sustainable Fisheries Management Project. (Accessed May 8, 2020).
857 https:/[/www.c](http://www.crc.uri.edu/download/GH2014_PGM187_CRC_FIN508.pdf)r[c.uri.edu/download/GH2014_PGM187_CRC_FIN508.pdf](http://www.crc.uri.edu/download/GH2014_PGM187_CRC_FIN508.pdf)
858 Dalheimer, B., Herwartz, H., & Lange, A , 2021. The threat of the oil market turmoils to food
859 price stability. Energy Economics, 93(2021) 105029.
860 Finegold C, Gordon A, Mills D, Curtis L,& Pulis A ., 2010. Western Region Fisheries Sector
861 Review. World Fish Center. USAID Integrated Coastal and Fisheries Governance
862 Initiative for the Western Region, Ghana, 1–84.
863 Fisheries Commission., 2022. Annual Performance Report. Ministry of Fisheries and
864 Aquaculture Development. Accra: Ministry of Fisheries and Aquaculture Development.
865 Food and Agricultural Organisation. 2022. The state of food security and nutrition in the world,
866 repurposing food, and agricultural politics to make healthy diets more affordable.
867 Food and Agricultural Organization (FAO). 2020. The State of World Fisheries and Aquaculture
868 2020. Sustainability in action. Rome. https://doi.org/10.4060/ca9229en
869 Freduah, G., Fidelman, P., & Smith, T. F. (2019). Adaptive capacity of small‐scale coastal 870	fishers to climate and non‐climate stressors in the Western region of Ghana. The 871	Geographical Journal, 185(1), 96–110.

| 872 | Ghana Statistical Service., 2014. Ghana Living Standards Survey 6, Main |  |
| --- | --- | --- |
| 873 | Repor[t.h](http://www.statsghana.gov.gh/gssmain/fileUpload/Living%20conditions/GLSS6_)tt[p://www.statsghana.gov.gh/gssmain/fileUpload/Living%20conditions/GLSS6_](http://www.statsghana.gov.gh/gssmain/fileUpload/Living%20conditions/GLSS6_) |  |
| 874 | Main%20Report.pdf |  |
| 875 | Ghana Statistical Services, 2020. Statistical Bulletin, Consumer Price |  |
| 876 | Index.https:/[/www.sta](http://www.statsghana.gov.gh/gssmain/fileUpload/Price%20Indices/Bulletin_)t[sghana.gov.gh/gssmain/fileUpload/Price%20Indices/Bulletin_%20](http://www.statsghana.gov.gh/gssmain/fileUpload/Price%20Indices/Bulletin_) |  |
| 877 | CPI%20August%202022.pdf |  |
| 878 | GhanaWeb, (2022). Year in review: Here is the number of times fuel prices were increased in |  |
| 879 | 2022. [Year in Review: Here is the number of times fuel prices were increased in 2022](https://www.ghanaweb.com/GhanaHomePage/business/Year-in-Review-Here-is-the-number-of-times-fuel-prices-were-increased-in-2022-1684070) |  |
| 880 | [(ghanaweb.com)](https://www.ghanaweb.com/GhanaHomePage/business/Year-in-Review-Here-is-the-number-of-times-fuel-prices-were-increased-in-2022-1684070) |  |
| 881 | Government of Ghana, 2019. The Budget Statement of the Government of Ghana for the 2018 |  |
| 882 | Financial Year. (Accessed June 6, 2021). |  |
| 883 | [https://www.mofep.gov.gh/sites/default/files/budget-statements/2019-Budget-Statement-](https://www.mofep.gov.gh/sites/default/files/budget-statements/2019-Budget-Statement-and-Economic-Policy_.pdf) |  |
| 884 | [and-Economic-Policy_.pdf](https://www.mofep.gov.gh/sites/default/files/budget-statements/2019-Budget-Statement-and-Economic-Policy_.pdf) |  |
| 885 |  | **Formatted:** Indent: Left: 0", First line: 0" |
| 886 | Hay, I. (2010). Qualitative research methods in human geography. Oxford Univeristy Press. |  |
| 887 | Hasselberg A E, Aakre I, Scholtens J, Overa J, Kolding J, Bank MS, Atter A,& Kjellevold M |  |
| 888 | 2020. Fish for food and nutrition security in Ghana: Challenges and opportunities. Global |  |
| 889 | Food Security, 26, 100380. |  |
| 890 | Kamasa, K, Amposah, B.D., & Forson, P. 2020. Do crude oil price changes affect economic |  |
| 891 | welfare? Empirical evidence from Ghana. Ghana Mining Journal, 20(1), 51-58. |  |
| 892 | Koranteng, K, A., 2022. China’s capture of Ghana’s fishing industry threatening food security. |  |
| 893 | [https://www.myjoyonline.com/chinas-capture-of-ghanas-fishing-industry-threatening-](https://www.myjoyonline.com/chinas-capture-of-ghanas-fishing-industry-threatening-food-security/) |  |
| 894 | [food-security/.](https://www.myjoyonline.com/chinas-capture-of-ghanas-fishing-industry-threatening-food-security/) |  |
| 895 | Koranteng, K.A., Ofori-Danson, P.K., & Entsua-Mensah, M , 2000. Fish and fisheries of the |  |
| 896 | Muni lagoon in Ghana, West Africa. Biodiversity and Conservation, 9, 487-499. |  |
| 897 | Kwadzo M .2022. Effects of the Closed Fishing Season on the Livelihood of Fisheries Workers: |  |
| 898 | A Case Study of Fisheries Workers in Elmina in the Central Region of Ghana.” World |  |
| 899 | Journal of Agricultural Research, 10(4):102-110 |  |
| 900 | Lazar N, Yankson K, Blay J, Ofori-Danson P, Markwei P, Agbogah K, Bannerman P, Sotor M, |  |
| 901 | Yamoah KK, & Bilisini WB,2016. Rebuilding Depleted Small Pelagic Stocks in Ghana. |  |
| 902 | A Closed Fishing Season Proposal to the Ministry of Fisheries and Aquaculture |  |
| 903 | Development. The Scientific and Technical Working Group. The USAID/Ghana |  |
| 904 | Sustainable Fisheries Management Project (SFMP). Narragansett, RI: Coastal Resource |  |
| 905 | Center, Graduate School of Oceanography, University of Rhode Island. |  |

906	March A, & Failler P, 2022. Small-scale fisheries development in Africa: Lessons learned and

907	best practices for enhancing food security and livelihoods. Marine Policy, 136, 104925.

908	Marcusi E. D, Rafon J.K A,& Marcusi E. S, 2022. Impact of Covid 19 and closed fishing season

909	on commercial fishers of Davao Gulf, Mindanao, Philippines. Ocean and Coast

910	Management, 217, 105997.

911	Marquette C. M, Koranteng K. A, Overå R, & Bortei-Doku A. E 2002. Small-scale fisheries, 912	population dynamics, and resource use in Africa: The case of Moree, Ghana. Ambio 31, 913	324–336.

914 Miani, A.M., Dehkordi, M.K., Narges, S., Lassois, L., Rong, T., & Hossein, A (2023). Toward
915 sustainable rural livelihoods approach: Application of grounded theory in Ghazni
916 province, Afghanistan. Applied Geography, 154, 102915.
917 Ministry of Finance., 2019. Ghana's Oil Production Estimated to go up to 500,000 per day by
918 2024 https://mofep.gov.gh/press-release/2019-02-14/ghana%27s-oil-production-
919 estimated-to-go-up-to-500%2C000-Barrels-per-day-by-2024
920 Ministry of Fisheries & Aquaculture Development, 2015. Fisheries Management Plan of Ghana. 921	A National Policy for Managing the Marine Fisheries Sector 2015-2019 56. Retrieved 922	from https://mofad.gov.gh/fisheries-management-plan-of-ghana- 2015-2019.

923	Ministry of Fisheries & Aquaculture Development., 2016. Ghana marine canoe frame survey. 924	Information report no.35. Fisheries Scientific Survey Division, Fisheries Commission, 925	Ghana.

926 Munro, G., & Sumaila, U. R. (2002). The impact of subsidies upon fisheries management and
927 sustainability: the case of the North Atlantic. Fish and Fisheries, 3(4), 233-250.
928 Natarajan, N., Newsham, A., Rigg, J & Suhardiman, D, 2022. A sustainable livelihoods
929 framework for the 21st century. World Development, 155, 105898.
930 Natural Resource Governance Institute, 2020. Ghana’s oil sales. Using commodity trading data 931	for accountability. [https://resourcegovernance.org/sites/default/files/documents/ghana-](https://resourcegovernance.org/sites/default/files/documents/ghana-oil-sales-using-commodity-trading-data-for-accountability.pdf) 932	[oil-sales-using-commodity-trading-data-for-accountability.pdf](https://resourcegovernance.org/sites/default/files/documents/ghana-oil-sales-using-commodity-trading-data-for-accountability.pdf)

933	Nchor, D., Klepac, V., & Adamec, V. 2016. Effects of oil price shocks on the Ghanaian

934	economy. Acta Universitatis Agricultural et Silviculture Mendelianae Brunensis, 64(1):

935	315-324.

936	Nolan, C. 2019. Power and Access Issues in Ghana’s Coastal Fisheries: A Political Ecology of A

937	Closing Commodity Frontier. Marine Policy 108:103621

938	Nowell, L.S., J.M. Norris, D.E. White, & N.J. Moules. (2017). Thematic analysis: Striving to 939	meet the trustworthiness criteria. International Journal of Qualitative Methods 16 (1): 940	1609406917733847

941	Obeng-Odoom, F. (2014). Black gold in Ghana: crude days for fishers and farmers? Local

942	Environment, 19(3), 259–282. https://doi.org/10.1080/13549839.2013.788481

943	Ocloo D. R. (2022). Fish crisis at Tema Harbour: Only 4 of 76 commercial fishing vessels are 944	functional. Graphic Online. [https://www.graphic.com.gh/news/general-news/fish-crisis-](https://www.graphic.com.gh/news/general-news/fish-crisis-at-tema-harbour-only-4-of-76-commercial-fishing-vessels-functional.html) 945	[at-tema-harbour-only-4-of-76-commercial-fishing-vessels-functional.html](https://www.graphic.com.gh/news/general-news/fish-crisis-at-tema-harbour-only-4-of-76-commercial-fishing-vessels-functional.html)

946	Ofori-Danson, P.K., Asiedu, B., Amponsah, S.K.K., & Crawford, B. (2019). Assessment of the 947	Socioeconomic, Food Security, and Nutrition Impacts of the 2019 Canoe Fishery Closed 948	Fishing Season in Ghana. USAID/Ghana Sustainable Fisheries Management Project.

949 Narragansett, RI: Coastal Resources Center, Graduate School of Oceanography,
950 University of Rhode Island. GH2014_SCI076_CRC. 109 pp
951 Oku, E. (2018). Effect of world oil prices on Ghana's cereal prices. Master’s degree Dissertation.
952 University of Ghana.
953 Okyere, E.Y., Adu-Boahen, K., Boateng, I., Dadson, I.Y., Boanu, N. Y & Kyeremeh, S. 2023. 954	Analysis of ecological health status of the Muni Lagoon: Evidence from heavy metal 955	content in its water and fish samples. Geography and environment, 10, e00115.

956	Okyere, I., Chuku, E. O., Ekumah, B., Angnuureng, D. B., Boakye-Appiah, J. K., Mills, D. J., ... 957	& Crawford, B. 2020. Physical distancing and risk of COVID-19 in small-scale fisheries: 958	a remote sensing assessment in coastal Ghana. Scientific reports, 10(1), 1–13.

959	Owusu V & Andriesse E, 2020. From free access regime to the closed fishing season: Lessons 960	from small-scale coastal fisheries in the Western Region of Ghana. Marine Policy, 121, 961	104162.

962	Owusu V, & Andriesse E , 2022. Local differentiation and adapting to climate change in coastal

963	Ghana. Geographical Review. DOI: 10.1080/00167428.2021.2023530.

964	Owusu, V & Adjei, M., 2021. Politics, power, and unequal access to fisheries subsidies among 965	small-scale coastal fisherfolk in Ghana. Ocean and Coastal Management, 214 (2021) 966	10592.

967	Owusu, V., Tamatey, E.L, Adjei, M, Ogbe, M .2023. Impact of offshore petroleum extraction 968	and ‘ocean grabbing’ on small-scale fisheries and coastal livelihoods in Ghana. Journal of 969	Maritime Studies.22(17)

970	Owusu., V., 2019. Impacts of the petroleum industry on the livelihoods of fisherfolk in Ghana: 971	A case study of the Western Region. The Extractive Industries and Society, 6(4), 1256– 972	1264.

973 Parker, W., R., & Tyedmers, P., H. (2015). Fuel consumption of global fishing fleets: current
974 understanding and knowledge gaps. Fish and Fisheries, pp. 16, 684–696.
975 Penney, R., Wilson, G., & Rodwell, L. (2017). Managing Sino-ghanaian fishery relations: A
976 political ecology approach. Marine Policy, pp. 79, 46–53.
977 Perry, R.I., Sumaila, U.R., 2007. Marine ecosystem variability and human community responses:
978 The example of Ghana, West Africa. Mar. Policy 31, 125–134.
979 Sasu, D.D.(2023). Weekly prices of gasoline in Ghana from October 19, 2020, to April 17, 2023.
980 <https://www.statista.com/statistics/1200108/weekly-gasoline-prices-in-ghana/>
981 Scoones, I. (2009). Livelihoods perspectives and rural development. Journal of Peasant Studies,

982	36(1), 171–196.

983	Serrat, O. (2008). The sustainable livelihoods approach. Regional & Sustainable Development

984	Department, Asian Development. Manila. The Philippines.

985	Sumaila. U.R, The. L., Watson, R., Tyedmers, P., Pauly, D. 2008. Fuel price increase, subsidies, 986	overcapacity, and resource sustainability, ICES Journal of Marine Science, 65(6), 832– 987	840.

988 World Bank,2016. Safety and sustainability for small-scale fishers in West Africa.
989 https:/[/www](http://www.worldbank.org/en/news/feature/2016/05/16/safety-and-sustainability-for-).[worldbank.org/en/news/feature/2016/05/16/safety-and-sustainability-for-](http://www.worldbank.org/en/news/feature/2016/05/16/safety-and-sustainability-for-)
990 small-scale-fishers-in-west-africa.
991 World Bank.2023 World Bank Country and Lending
992 Groups.https://datahelpdesk.worldbank.org/knowledgebase/articles/906519
993 Yang, J., Owusu, V., Andriesse, E., & Dziwornu Ablo, A. 2019. In-situ adaptation and coastal 994	vulnerabilities in Ghana and Tanzania. The Journal of Environment & Development, 995	28(3), 282-308.

996

Response to Reviewers

**Effect of rising fuel prices on small-scale fisheries livelihoods and marine sustainability in Ghana.**

##### Academic editor

1. Please ensure that your manuscript meets PLOS ONE's style requirements, including those for file naming. The PLOS ONE style templates can be found at

##### Response

The manuscript has been formatted in relation to PLOS ONEs style requirements.

2. Note from Emily Chenette, Editor in Chief of PLOS ONE, and Iain Hrynaszkiewicz, Director of Open Research Solutions at PLOS: Did you know that depositing data in a repository is associated with up to a 25% citation advantage (https://doi.org/10.1371/journal.pone.0230416)? If you’ve not already done so, consider depositing your raw data in a repository to ensure your work is read, appreciated and cited by the largest possible audience. You’ll also earn an Accessible Data icon on your published paper if you deposit your data in any participating repository ([https://plos.org/open-science/open-data/#accessible-](https://plos.org/open-science/open-data/#accessible-data) [data](https://plos.org/open-science/open-data/#accessible-data)).

**Response**

Thank you for the information

3. You indicated that ethical approval was not necessary for your study. We understand that the

framework for ethical oversight requirements for studies of this type may differ depending on the setting and we would appreciate some further clarification regarding your research. Could you please provide further details on why your study is exempt from the need for approval and confirmation from your

institutional review board or research ethics committee (e.g., in the form of a letter or email

correspondence) that ethics review was not necessary for this study? Please include a copy of the correspondence as an ""Other"" file.

##### Response

Over the past few years l have established working relationships with fisherfolks in the Central Region of Ghana and has built trust with the communities and leadership of the fisherfolks for successful work. The already established relationship means easy entry into the communities and being affiliated with an academic community gives the team credibility to conduct ethically appropriate research with the rights and safety of the research participants and the researcher intact. Thus, the collaboration between the researchers and the case study communities in the Central Region of Ghana has developed into a long-standing respectful and reciprocal relationship.

An introductory letter has also been attached File.

5. PLOS requires an ORCID iD for the corresponding author in Editorial Manager on papers submitted after December 6th, 2016. Please ensure that you have an ORCID iD and that it is validated in Editorial Manager. To do this, go to ‘Update my Information’ (in the upper left-hand corner of the main menu),

and click on the Fetch/Validate link next to the ORCID field. This will take you to the ORCID site and allow you to create a new iD or authenticate a pre-existing iD in Editorial Manager. Please see the following

video for instructions on linking an ORCID iD to your Editorial Manager account: <https://www.youtube.com/watch?v=_xcclfuvtxQ>.

**RESPONSE**

ORCID iD 0009-0004-4801-0985

[0009-0004-4801-0985](https://orcid.org/0009-0004-4801-0985?lang=en)

6. Please amend your manuscript to include your abstract after the title page. Thank you, this has been done
7. We note that Figure 4 in your submission contain [map/satellite] images which may be copyrighted. All PLOS content is published under the Creative Commons Attribution License (CC BY 4.0), which means

that the manuscript, images, and Supporting Information files will be freely available online, and any

third party is permitted to access, download, copy, distribute, and use these materials in any way, even commercially, with proper attribution. For these reasons, we cannot publish previously copyrighted maps or satellite images created using proprietary data, such as Google software (Google Maps, Street View, and Earth). For more information, see our copyright guidelines:

[http://journals.plos.org/plosone/s/licenses-and-copyright.](http://journals.plos.org/plosone/s/licenses-and-copyright)

We require you to either (1) present written permission from the copyright holder to publish these figures specifically under the CC BY 4.0 license, or (2) remove the figures from your submission:

###### RESPONSE

Fig 4 has been removed.

8. Please review your reference list to ensure that it is complete and correct. If you have cited papers that have been retracted, please include the rationale for doing so in the manuscript text, or remove these references and replace them with relevant current references. Any changes to the reference list

should be mentioned in the rebuttal letter that accompanies your revised manuscript. If you need to cite a retracted article, indicate the article’s retracted status in the References list and also include a citation and full reference for the retraction notice.

###### RESPONSE

All the necessary changes have been effected. Thank you

##### Reviewer 2 Comments

1. The abstract clearly outlines the study's objective, methodology, key findings, and implications. However, it could be more concise, focusing on the most critical points. Anyway I suggest that author must reduce redundancy, such as "The study applied a mixed-methods approach to investigate the effects of fuel price hikes on the livelihoods of coastal fisherfolk using the sustainable livelihoods framework" can be shortened. Also authors need to highlight the main findings more succinctly, possibly breaking them into bullet points for clarity

##### Response

I thank the reviewer for pointing this out. The necessary improvements have been made.

2. The introduction provides a thorough background on the issue, including relevant statistics and references. The context of global oil prices affecting local economies is well established. Also the Sustainable Livelihoods Approach (SLA) is well explained and relevant to the study. However, I think 1 and 2 should be combined as "Introduction". I think authors need to work really hard to restructure this two sections.

I also suggested that this section is dense with information. Please consider breaking it into smaller paragraphs for better readability. Also author need to clearly state the research questions earlier in the introduction to guide the reader. Please also add more recent studies to support the discussion on SLA and its application to fisheries.

##### Response

Section 1 and 2 have been combined as introduction. The section has also been broken into smaller paragraphs to make it easy to read. All the other necessary corrections has been made.

3. I think authors need to provide more details on the sampling techniques and why 220 households and 20 stakeholders were chosen. Also author need to discuss any limitations of the methods used and how they were addressed.

##### Response

The sampling techniques has been further explained see page 13 -14.

The sample size used for the study is actually 320 not 220 as initially reported by the author. It’s a typological error, kindly pardon me. The process leading to the selection of the 320 participants is explained in pages 13-15.

4. I request the authors please use more visual aids like graphs and tables to summarize key data points. Also authors must provide more direct quotes from interviews to support qualitative findings.

##### Response

Thank you for this comment, l have worked on the comments accordingly , a new chart has been added and some interview quotations have also been added.

5. Please ensure that each finding is discussed in relation to the research questions. I think authors need to highlight the practical implications of the findings for policymakers and stakeholders more explicitly. Additionally authors need to include specific recommendations for future research, and address the limitations of the study briefly and suggest how future studies could overcome these.

##### Response

Thank you for this valuable comment. The discussion section has been worked on. Kindly see the discussion section.

6. Frankly speaking, this work lacks of any interesting point and to really sure what is a really new findings from the work. Please emphasize that and explain more carefully.

##### Response

This paper contributes to the literature on global fuel price hikes and its implications for marine fisheries sustainability by investigating the effects of oil price shocks on fisheries’ livelihoods in SSF communities in Ghana. The study considers the impacts and the various coping strategies fisherfolk deploy in response to fuel price shocks. The study contributes to the literature on coastal livelihoods, ocean governance, and natural resources management. Ghana is both an oil importer and exporter; hence, examining the effects of escalating global oil prices on the local fishing industry would interest the government, development practitioners, and policymakers. The results from the study could further help to devise a more specific stimulus package tailored to cater to the unique needs of the sector involved.
