# Supplementary material for: Effect of rising fuel prices on small-scale fisheries livelihoods and marine sustainability in Ghana
Source: PLoS One. 2025 Jan 13;20(1):e0317260. doi: 10.1371/journal.pone.0317260 (PMC11729924; doi:10.1371/journal.pone.0317260)
Supplement: Attachment — Submitted filename: Response to reviwer comments.docx [file pone.0317260.s013.docx]

**Effect of rising fuel prices on small-scale fisheries livelihoods and marine sustainability in Ghana.**

**1.**This should be part of the recommendation drawn from your study. It should not be included here as a specific objective

**Response**

I thank the reviewer for this important comment, I have deleted objective 3 accordingly.

2. Please briefly include the livelihood supported by the sector i.e. both direct and indirect/families beneficiary that relay on the aquatic resources.

**Response**

I thank the reviewer for this observation. The fisheries sector directly support the livelihoods of men and women in the coastal communities. The men are engaged in fishing , the women are involved in processing and trading of fish. Indirectly the sector also support the livelihoods of boat builders, food vendors etc Please page 9, line 226-230.

3. Why the interview was conducted with stakeholders only? It should have been conducted with selected fishers too, to elaborate the quantitative finding.

**Response**

The stakeholders includes fisherfolk, government officials and NGOs. This is further explained – please see page 11, line 287-289.

4. Please compare the change in fuel price/a single production of the fishers before and after the fuel price change in GHANA

**Response**

Thank you, this has been done.

5.If you had employed quantitative survey, why you did not measure the livelihoods of the fishers using indicators for these different capital assets.

**Response**

I thank the reviewer for this observation. Unfortunately, l did not measure the livelihood of the fishers in relation to the capital assets due to the limited scope of study. Since the study is limited in its scope l decided to focused on describing how the fuel price hikes have negatively affected the livelihood assets of fisherfolk.

6. If you have a data, please include one table that indicate fuel consumption of fishers/trip, boat horsepower with their respective fuel consumption, and enquiring cost per production time and production in tone/kg. The profitability to support their livelihood should be related with their income and expense

**Response**

Different boats catch different species using boat engine capacity of 40hp. In addition, the number of fishing days differ from boat to boat. For instance the quantity of fish for a set net gear will differ from that beach seine because they all target different species and the weight differ in terms of the fish. So the average fish catch for 40hp per trip will be difficult to provide.

I have however included information provided by one of the interviewed canoe owner in relation to fuel consumption per fishing trip with 40 hp / cost of production and volume of fish landed in kg. Please see page 17, line 384 -393.

7. Please include some points on sustainability either here or in recommendation section

**Response**

This have been done, please see page page 25 , line 617.

8. Please remove the reference from this section. As this is your conclusion and recommendation from the study.

**Response**

Thank you, this has been done.
